# Self-Assembled Lanthanide Antenna Glutathione Sensor for the Study of Immune Cells

**DOI:** 10.1021/acssensors.1c02439

**Published:** 2022-01-15

**Authors:** Francisco Fueyo-González, Laura Espinar-Barranco, Rosario Herranz, Ibon Alkorta, Luis Crovetto, Miguel Fribourg, Jose Manuel Paredes, Angel Orte, Juan A. González-Vera

**Affiliations:** † Instituto de Química Médica (CSIC), Juan de la Cierva 3, 28006 Madrid, Spain; ‡ Nanoscopy Laboratory, Departamento de Fisicoquímica, Unidad de Excelencia de Química Aplicada a Biomedicina y Medioambiente, Facultad de Farmacia, Universidad de Granada, Campus Cartuja, 18071 Granada, Spain; § Department of Medicine, Translational Transplant Research Center, Immunology Institute, Icahn School of Medicine at Mount Sinai, New York, New York 10029, United States

**Keywords:** glutathione, luminescent sensor, self-assembled antenna, lanthanide, time-resolved luminescence, flow cytometry, T cells, T_REG_

## Abstract

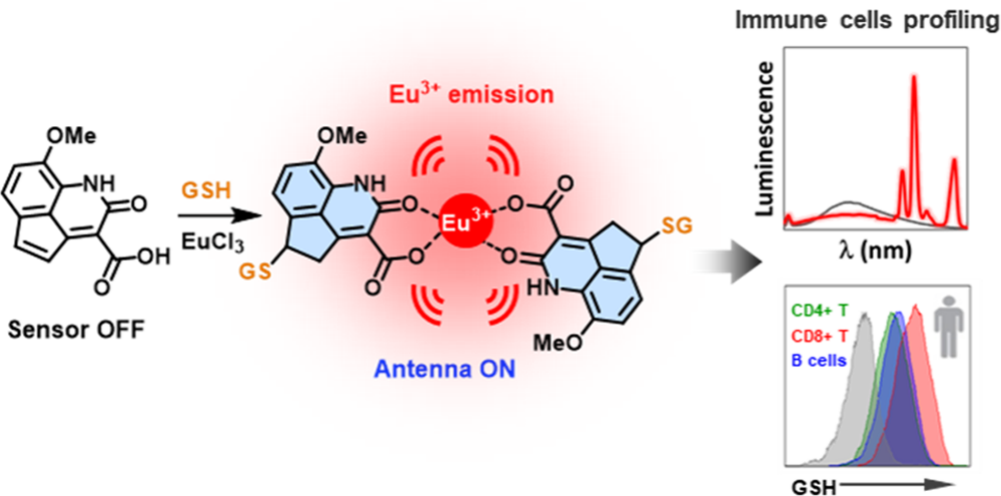

The small molecule 8-methoxy-2-oxo-1,2,4,5-tetrahydrocyclopenta[de]quinoline-3-carboxylic acid (**2b**) behaves as a reactive non-fluorescent Michael acceptor, which after reaction with thiols becomes fluorescent, and an efficient Eu^3+^ antenna, after self-assembling with this cation in water. This behavior makes **2b** a highly selective GSH biosensor, which has demonstrated high potential for studies in murine and human cells of the immune system (CD4^+^ T, CD8^+^ T, and B cells) using flow cytometry. GSH can be monitored by the fluorescence of the product of addition to **2b** (445 nm) or by the luminescence of Eu^3+^ (592 nm). **2b** was able to capture baseline differences in GSH intracellular levels among murine and human CD4^+^ T, CD8^+^ T, and B cells. We also successfully used **2b** to monitor intracellular changes in GSH associated with the metabolic variations governing the induction of CD4^+^ naïve T cells into regulatory T cells (T_REG_).

Biologically active thiols known as biothiols, which include cysteine (Cys), homocysteine (Hcy), glutathione (GSH), and hydrogen sulfide (H_2_S), play a central role in the intracellular regulation of redox homeostasis and in the maintenance of cellular functions, such as post-translational modifications, biocatalysis, metal binding, and xenobiotic detoxification.^1,2^ Oxidative stress is a key feature of a wide variety of chronic and degenerative diseases, and changes in the levels of biothiols have been associated with various diseases.^3−8^ Distinct responses to metabolic stimuli (bioenergetic signatures) have been associated with differences in the immune function.^9,10^ In recent years, several studies have shed light on the dynamic and sophisticated connection between metabolic programs and the function of specialized cells in the immune system.^10,11^ This crucial role of metabolism in the control of immune processes, including inflammation, has led to the emergence of a new field of immunometabolism.^11−13^ It is increasingly recognized that biothiols play a key role in regulating the metabolic adaptability and thereby the function of cells of the immune system.^14−21^ One of the latest discoveries in this field is the regulation of functions through the synthesis and release of various biothiols, in particular, GSH, which affects the metabolism and function of the immune system’s effector cells.^12,17,21−24^ Consequently, the interest in developing tools to monitor biothiol levels in immune cells in clinical samples has grown exponentially. To this aim, diverse probes and techniques have been developed for the detection of biothiols. Among the methods used, those based on fluorescence emission are among those that provide the greatest advantages due to their simplicity, low detection limits, and ease of use.^25−28^ However, selective and sensitive methods to detect and monitor GSH in cells with flow cytometry, a fluorescence-based, gold-standard tool for the identification and classification of cellular populations, remain an unmet need in the immunology field. However, current methods to measure GSH lack selectivity and sensitivity, and their suitability to flow cytometry remains largely unexplored. The few that have been studied with this technique in immune cells include monochloro (bromo) bimane,^29^ mercury orange,^30^*o*-phthaldialdehyde, and chloromethyl fluorescein diacetate,^31^ but none of them is selective for GSH.^32^

Many probes for biothiol sensing are based on Michael acceptors, in which following a nucleophilic attack of the sulfhydryl group and its addition to a double bond of the probe, their fluorescence increases notably. Luminescent sensors based on lanthanide complexes present several advantages over classical organic fluorophores, such as a very high luminescence lifetime and narrow emission bands, which allow an increase in the sensitivity and signal-to-noise ratio, avoiding natural background fluorescence in time-resolved luminescence spectroscopy.^33−35^ Among the few lanthanide-based biothiol sensors reported in the literature,^36,37^ to our knowledge, no lanthanide antenna-based sensors, which self-assemble in water have yet been reported.

In this field, we have recently reported the discovery of the small and simple structure lanthanide antenna in organic solvents **1a** (Scheme 1).^38^ The lanthanide sensitization by **1a** is quenched by H_2_O addition, setting the basis for its demonstrated application as a H_2_O sensor. In our screening for potential lanthanide antennas, we observed that the free acid **1b**(39) (Scheme 1) was also able to sensitize the emission of Tb^3+^ and more extensively Eu^3+^ in H_2_O. For the design of a suitable biothiol sensor from **1b**, we synthesized the oxidized analogue 2-oxo-1,2-dihydrocyclopenta[*de*]quinoline-3-carboxylic acid, **2b**, which showed high Michael acceptor reactivity against thiols and an excellent fluorogenic behavior upon reaction. Herein, we report the design and synthesis of this free acid, the photophysical properties and lanthanide sensitization of **1b** and **2b**, the reactivity of **2b**, and proof-of-concept studies of the application of this biothiol sensor to study the cells of the immune system. Strikingly, the results described herein demonstrate that **2b** is a selective GSH sensor, which after its reaction with this biothiol, self-assembles in water with a lanthanide cation and, as an antenna, transfers its energy to the lanthanide ion resulting in the long-lived luminescence emission of the lanthanide.

**Scheme 1:**
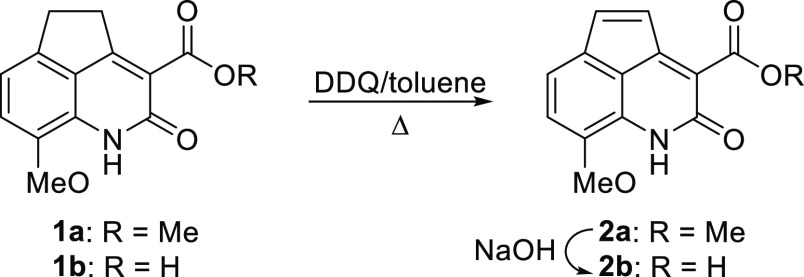
Synthesis of 8-Methoxy-2-oxo-1,2-dihydrocyclopenta[*de*]quinoline-3-carboxylic Acid (**2b**)

## Results and Discussion

### Synthesis of **2b**

As shown in Scheme 1, the free acid **2b** was obtained in good yield from methyl ester **1a** by oxidation to **2a**, with 2,3-dichloro-5,6-dicyano-1,4-benzoquinone (DDQ) in refluxing toluene, followed by saponification to the corresponding free acid **2b** by heating with 2 N NaOH.

### Photophysical Properties of **1b** and **2b**

The photophysical properties of **1b** and **2b** in CH_3_CN and H_2_O are shown in Table 1. The UV/visible spectrum of **1b** showed an absorption maximum at 320 nm, with a small shoulder at around 375 nm, while the oxidized analogue **2b** showed a broad absorption band centered around 390 nm, with no significant influence of the solvent polarity in both cases. As expected, due to the extension of the conjugation in the chromophore moiety, the absorption maximum of **2b** was shifted approximately 70 nm toward longer wavelengths when compared to that of **1b**. In fact, solutions of **1b** were colorless, while those of **2b** were orange as observed by the naked eye. Regarding emission properties, interestingly, oxidized compound **2b** showed almost negligible emission (Φ_F_ of **2b** was almost 20 times lower than that of **1b**).

**Table 1:** Photophysical Properties of Free Carboxylic Acids **1b** and **2b**

compd^a^	solvent	λ_max_^abs^ (nm)	ε (M^–1^ cm^–1^)	λ_max_^em^ (nm)	Φ_F_^b^
**1b**	CH_3_CN	320, 375	4720	450	0.09
	H_2_O	320, 373	5451	472	0.11
**2b**	CH_3_CN	392	2125	462	0.008
	H_2_O	390	3050	471	0.006

a Measured in duplicate at 12 μM concentration.

b Quantum yields calculated with reference to quinine sulfate (in 0.1 M H_2_SO_4_).

This low fluorescence emission of **2b** could be due to an antiaromatic character of its 2-oxo-1,2-dihydrocyclopenta[*de*]quinoline [4*n*]π-electron system, according to Hückel’s rules.^40−42^ To clarify this hypothesis, TD-DFT calculations were carried out with the B3LYP functional and the 6-31+G(d,p) basis set,^43−46^ within the Gaussian-16 package^47^ to determine the minimum energy structures of the free carboxylic acids **1b** and **2b** in their singlet ground energy state (S_0_) and in the excited states S_1_ and T_1_, and their respective harmonic oscillator model of aromaticity (HOMA) values^48−52^ for the common fused ring of 2-oxo-quinoline. Four tautomeric/rotamer structures were considered in the study of the geometries of the S_0_, S_1_, and T_1_ energy states of **1b** and **2b**, one with a keto group at position 2 (**1A** and **2A** in Figure S1 of the Supporting Information) and the other three with an enol group at that position (**1B**–**1D** and **2B**–**2D** in Figure S1). Calculations (Table S1) showed that keto tautomer **A** is the minimum energy form for both **1b** and **2b** in the ground state S_0_, and in the T_1_ state of **2b**, while the enol tautomer **D** was that of minimum energy in the excited states S_1_ and T_1_ of **1b** and in the S_1_ of **2b**, although in the latter case its energy was very near to that of keto tautomer **A** (1.6 kJ mol^–1^). These results indicate that excitation induces tautomerization in **1b** and **2b**. Calculations of the HOMA index values for the 2-oxo-quinoline-fused ring common in **1b** and **2b** showed lower values for **2b** than for **1a** in the three energy states S_0_, S_1_, and T_1_ (Table S2) and therefore, a lower aromatic character in **2b**. Interestingly, a small decrease in the aromatic character of the six-membered rings of acenaphthylene compared to naphthalene has been reported.^53^ The calculated HOMA values for the peripheral tricyclic skeleton of the 1,2-dihydrocyclopenta[*de*]quinoline system of **2b** (Table S3) indicated an aromatic character for the three energy states.

On the other hand, when comparing NMR data of the oxidized compound **2b** with those of **1b** (Table S4), the most significant changes with respect to aromaticity were a 0.21 ppm displacement of 7-H toward a higher field in **2b** with respect to that of **1b**, and the displacements of carbons C_3a_ (29.8 pm), C_8a_ (8.3 pm), and C_8b_ (11.8 pm) also toward a higher field in **2b** with respect to those of **1b**. These data are indicative of a decrease in the deshielding of the aromatic ring current in **2b** with respect to **1b** and, therefore, a lower aromatic character, which could explain their photophysical behavior.

The ability of compounds **1b** and **2b** to directly bind lanthanide ions in the H_2_O solution (54 μM) and sensitize their emission was spectroscopically analyzed by the addition of 1 and 2 equivalents of TbCl_3_, EuCl_3_, DyCl_3_, and SmCl_3_. As shown in Figure S2, the free carboxylic acid **1b** sensitized the luminescence of the cations Tb^3+^ and Eu^3+^ but preferably that of the ^5^D_4_ → ^7^F_6_ (490 nm) and ^5^D_4_ → ^7^F_5_ (540–550 nm) Tb^3+^ bands. However, under the same conditions, the oxidized free carboxylic acid **2b** only sensitized the luminescence of Eu^3+^ but with a much lower intensity than **1b** (Figure S3).

### **2b** Behaves as a Selective and Sensitive Biothiol Sensor

The photophysical properties according to the structure of **2b**; we hypothesized that this molecule could be a good Michael acceptor in particular against thiols. This hypothesis was confirmed by following the reaction of **2b** (5 μM) with Cys (500 μM) in HEPES buffer pH 7.4 by HPLC–MS. As shown in Figure S4, when Cys was added just at the time of injection, the product of the addition of Cys to the double bond of **2b** was rapidly detected (t_*R*_ = 4.25 min).

**Figure 1:**
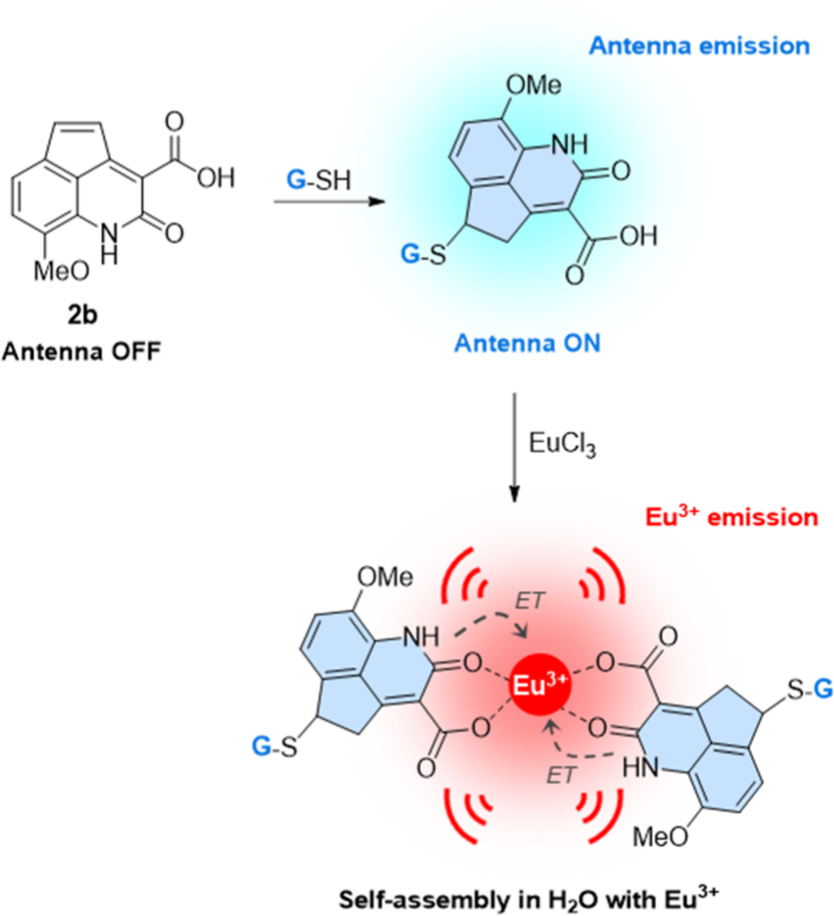
Schematic representation of biosensor **2b**. After the addition of GSH to Michael acceptor **2b**, the resulting antenna will increase its fluorescence. Moreover, if lanthanide ions are present, the antenna will self-assemble and intramolecularly transfer its energy (ET) to the metal, resulting in a significant increase in the red long-lived luminescence emission of Eu^3+^.

**Figure 2:**
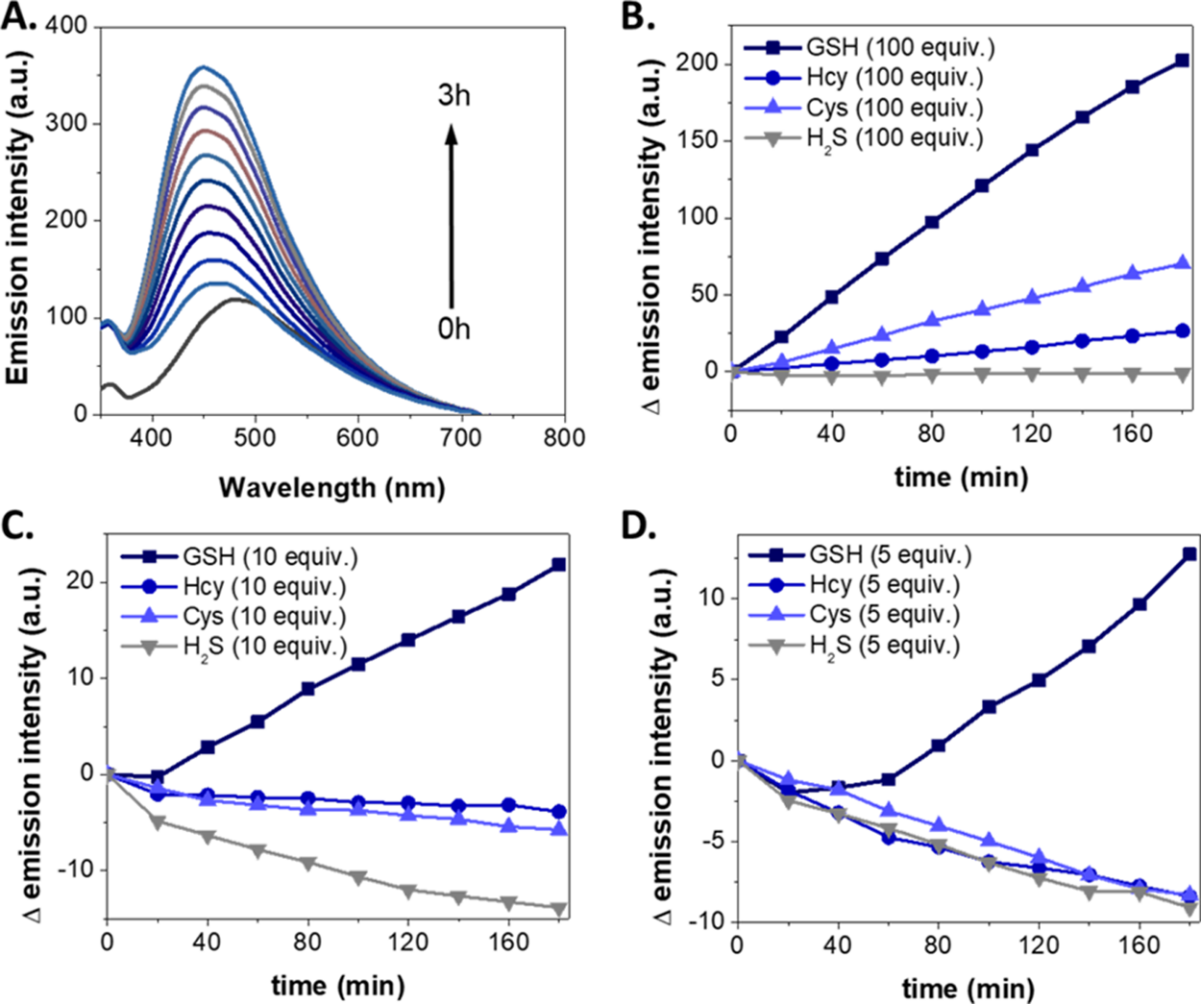
(A) Time-dependent fluorescence emission spectra of **2b** (5 μM, λ_ex_ = 320 nm) after the addition of 100 equiv of GSH. (B–D) Changes in the fluorescence emission intensity of **2b** (5 μM) at 445 nm (λ_ex_ = 320 nm) over time, after the addition of (B) 100, (C) 10, and (D) 5 equiv of GSH, Hcy, Cys, and H_2_S.

Considering the good reactivity of **2b** against Cys and the photophysical properties of **2b** and **1b**, we propose that **2b** could be employed as a fluorogenic biothiol sensor (Figure 1), and consequently we studied its time-dependent reactivity toward GSH, Hcy, Cys, and H_2_S in HEPES buffer (50 mM, pH 7.4) using luminescence spectroscopy. The addition of 100 equivalents of GSH to **2b** (5 μM) resulted in a notable fluorescence increase at 445 nm (λ_ex_ = 320 nm) with a reaction time from 0 to 3 h (Figure 2A). By contrast, upon the treatment of **2b** (5 μM) with 100 equivalents of Cys, Hcy, or H_2_S, this fluorescent increase was significantly lower (Figure 2B), which highlighted the selectivity of our sensor for GSH. This selectivity was confirmed upon the addition of 5 or 10 equiv of GSH, Hcy, Cys, and H_2_S, as only GSH led to a fluorescence increase (Figure 2C,D). Furthermore, no obvious changes were detected when other amino acids, such as Ala, or potential interferent species (H_2_O_2_ and Fe^2+^) were added (Figure S5), further emphasizing its selectivity toward thiols.

**Figure 3:**
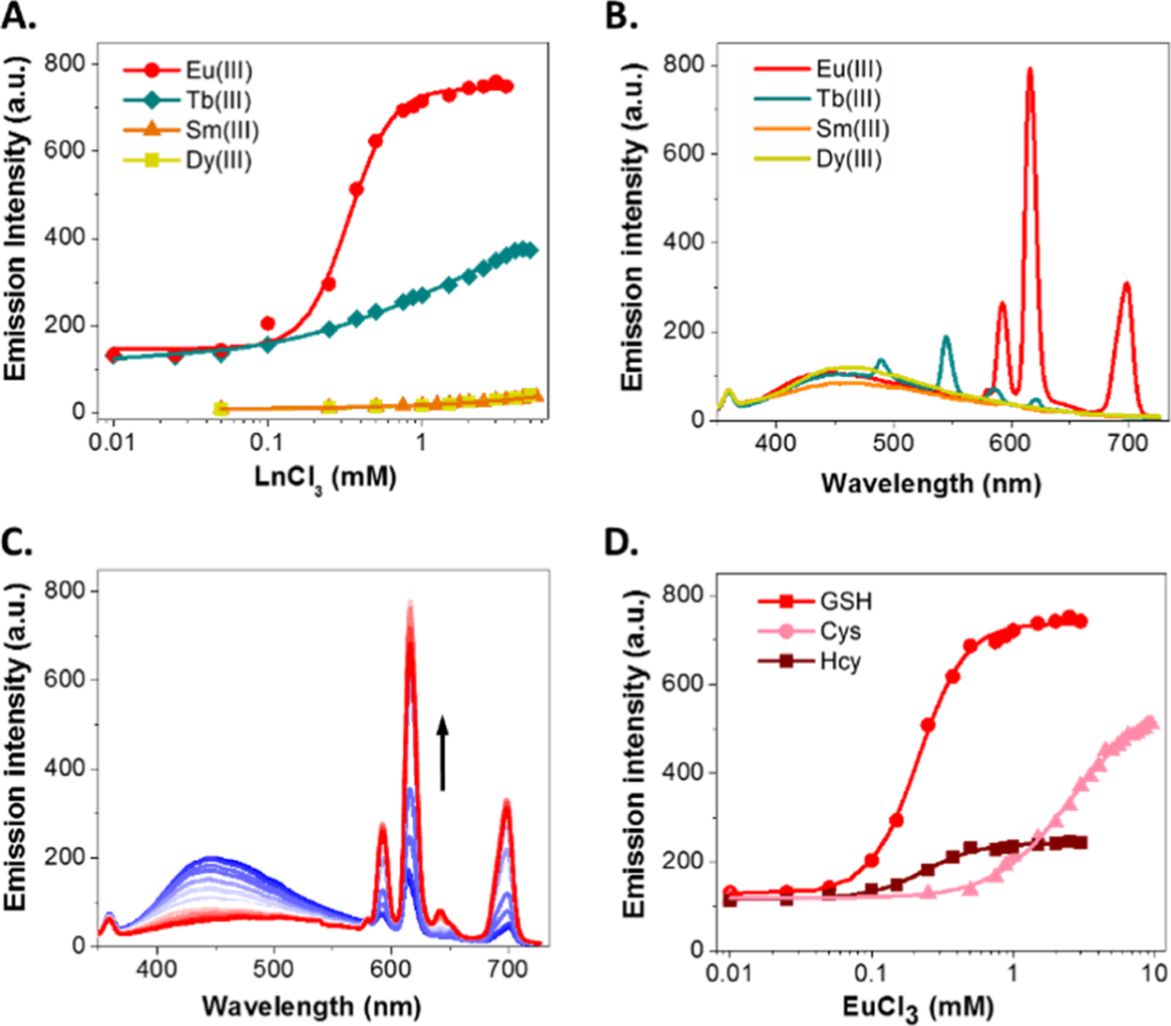
(A) Tb^3+^, Eu^3+^, Sm^3+^, and Dy^3+^ luminescence at their maximum emission wavelengths in the presence of **2b**-GSH (5 μM of **2b** and 100 equiv of GSH, λ_ex_ = 320 nm) as a function of the added EuCl_3_, TbCl_3_, DyCl_3_, and SmCl_3_ molar concentration (0.01–5.5 mM). (B) Emission spectra of **2b**-GSH (5 μM, λ_ex_ = 320 nm) after the addition of 100 equiv of EuCl_3_, TbCl_3_, DyCl_3_, and SmCl_3_. (C) Titration spectra of **2b**-GSH (5 μM, λ_ex_ = 320 nm) with increasing molar concentration of EuCl_3_ (0.025–3.0 mM, increase indicated by the arrow). (D) Eu^3+^ luminescence in the presence of reaction products **2b**-GSH, **2b**-Hcy, or **2b**-Cys (5 μM of **2b** and 100 equiv of biothiol, λ_ex_ = 320 nm) at 615 nm as a function of the added EuCl_3_ molar concentration (0.025–6.0 mM). Lines represent the fittings to a binding isotherm with a variable Hill slope equation model.

**Figure 4:**
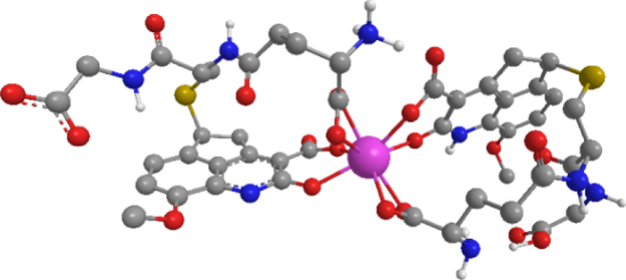
Proposed structure of the coordination of GSH-**2b** with Eu^3+^. The geometry of the europium complex has been optimized with the RM1 semiempirical method,^59,60^ as implemented in MOPAC2016.^61^

Given that **1b** acted as a suitable lanthanide antenna, an alternative strategy in the design of the biothiol sensor would entail the addition of lanthanide ions to the reaction product of the biothiol to **2b** (**2b**-SR) (Figure 1). To explore this strategy, we next studied the ability of product **2b**-GSH to directly bind lanthanide ions in the solution sensitizing their luminescence, thus resulting in a red-shifted fluorogenic sensing reaction and with extraordinary potential to apply time-gated luminescence analysis due to the long luminescence lifetime of lanthanide ions.^35^ We carried out the reaction of **2b** (5 μM) with 100 equivalents of GSH for 3 h (in HEPES 50 mM, pH 7.4), and then a titration of the corresponding addition product with increasing concentrations of TbCl_3_, EuCl_3_, DyCl_3_, and SmCl_3_ was performed (Figures 3A and S6). This led to the appearance of significant bands of the sensitized luminescence of the lanthanide cation, mainly the 5D^0^ → 7F^2^ Eu^3+^ band at 615 nm and, in a lower extent, the ^5^D_4_ → ^7^F_6_ (490 nm) and ^5^D_4_ → ^7^F_5_ (540–550 nm) Tb^3+^ bands (Figure 3B). The luminescence lifetimes (τ) of the Eu^3+^ and Tb^3+^ emissions for their complexes with **2b**-GSH were 122 ± 5 and 350 ± 1 μs, respectively, indicating great potential to use time-resolved and time-gated analyses in the detection of biothiols. On the other hand, we also prepared the addition products of Hcy, Cys, or H_2_S to **2b** (**2b**-Hcy, **2b**-Cys, and **2b**-H_2_S) and performed a titration with increasing concentrations of EuCl_3_. Compared to **2b**-GSH, which showed a significant energy transfer to the metal (Figure 3C), Eu^3+^ titration curves of **2b**-Hcy, **2b**-Cys, and **2b**-H_2_S led to a modest or negligible luminescent increase (Figures 3D and S7). Consequently, the τ of the Eu^3+^ emission for the complex of **2b**-GSH was higher than the ones of the complexes **2b**-Hcy and **2b**-Cys (Figure S8). The experimental data of the titrations fitted adequately to a binding isotherm with a variable Hill slope (see the Supporting Information for details). The fittings provided values for apparent microscopic dissociation constants of 0.213 ± 0.005, 0.235 ± 0.013, and 2.493 ± 0.092 mM, obtained for **2b**-GSH, **2b**-Hcy, and **2b**-Cys, respectively (Figure 3D). This confirmed the preference of Eu^3+^ to directly assemble **2b**-GSH or **2b**-Hcy and with much less affinity to **2b**-Cys. However, the higher Eu^3+^ luminescence intensity and lifetime exhibited by **2b**-GSH indicate more effective protection against quenching caused by water molecules in the complex with **2b**-GSH than with **2b**-Hcy.^54,55^ This protection of the lanthanide ion in **2b**-GSH is probably favored by the carboxylate group of the Glu residue present in GSH, which could aid in the formation of an extended coordination cage with the ion.^56−58^ To demonstrate this, the geometry of a proposed structure of the europium complex with two units of **2b**-GSH has been optimized with the RM1 semiempirical method (Figure 4).

**Figure 5:**
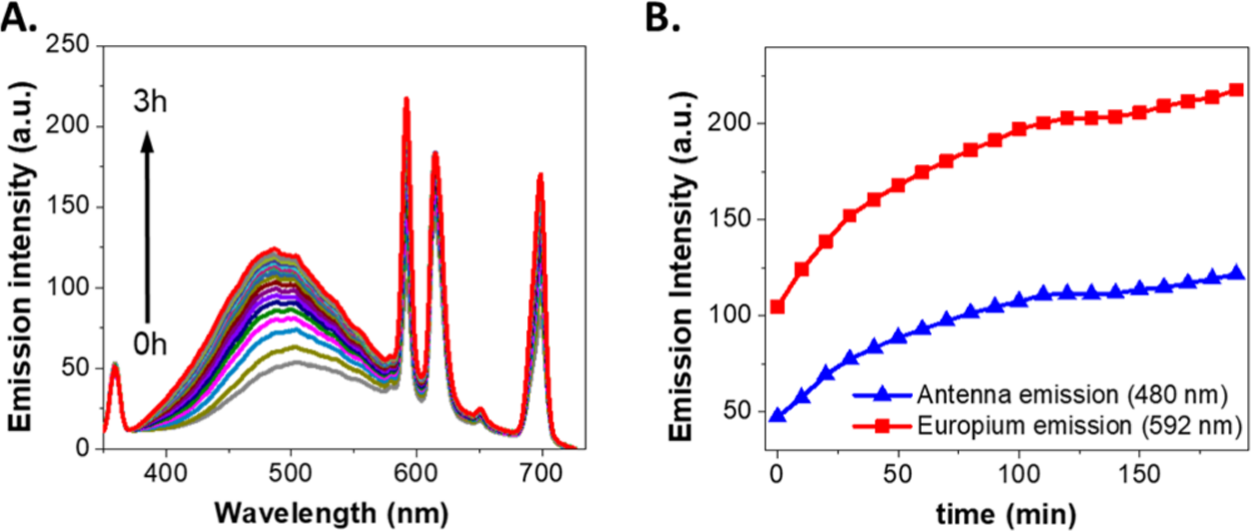
(A) Time-dependent luminescence emission spectra of **2b** (5 μM, λ_ex_ = 320 nm) in the presence of EuCl_3_ (1.5 mM) after the addition of 100 equiv of GSH. (B) Corresponding luminescence intensity at the emission of the antenna (480 nm, blue symbols) and Eu^3+^ (592 nm, red symbols).

The greater reactivity of **2b** with GSH and the greater sensitization of the Eu^3+^ luminescence would enhance the selectivity of the sensor for GSH in subsequent cell studies, apart from the much higher intracellular concentration of GSH (1–10 mM) than that of Cys or Hcy (30–200 μM).^62−64^ We thus focused our attention on the reactivity of our sensor **2b** against GSH (**2b**-GSH) and the selective sensitization of Eu^3+^ ions. We studied the reaction kinetics for the addition of 100 equivalents of GSH to **2b** (5 μM, HEPES 50 mM, pH 7.4) in the presence of EuCl_3_ (1.5 mM). This led to a significant luminescence increase (λ_ex_ = 320 nm) in both the emission of the antenna (at 480 nm) and Eu^3+^ (Figure 5). Because Eu^3+^ ions are added at the beginning of the reaction, they can be pre-coordinated with an unreacted **2b** probe. This may slightly change the surroundings of the lanthanide ions upon reaction, resulting in different areas of the 5D^0^ – 7F^1^ (592 nm) and 5D^0^ – 7F^2^ (616 nm) Eu^3+^ bands, when compared to the situation in which the lanthanide ions are added after the completion of the reaction. These results open the door to the use of **2b** as a self-assembled europium sensitizer to selectively report on the levels of GSH in real time, allowing the reaction to be monitored *in situ* at different wavelengths, broadening the palette for multiplexing applications. Nevertheless, we recommend using the 592 nm Eu^3+^ emission band because its magnetic dipole nature makes it less sensitive to the environment.^65^ The initial rates, obtained by analyzing the enhancement of the luminescence intensity at 592 nm, exhibited an excellent linear relationship with the initial concentration of GSH (in a logarithmic scale) (Figure S9). The linear fitting yielded a slope of 0.52 ± 0.07. This means a reaction order of 1/2 with respect to GSH, which indicates that the reaction mechanism is complex, possibly involving reversibility.^66,67^

### **2b** Can Be Used to Monitor Intracellular GSH Levels in Murine and Human Immune Cells

**Figure 6:**
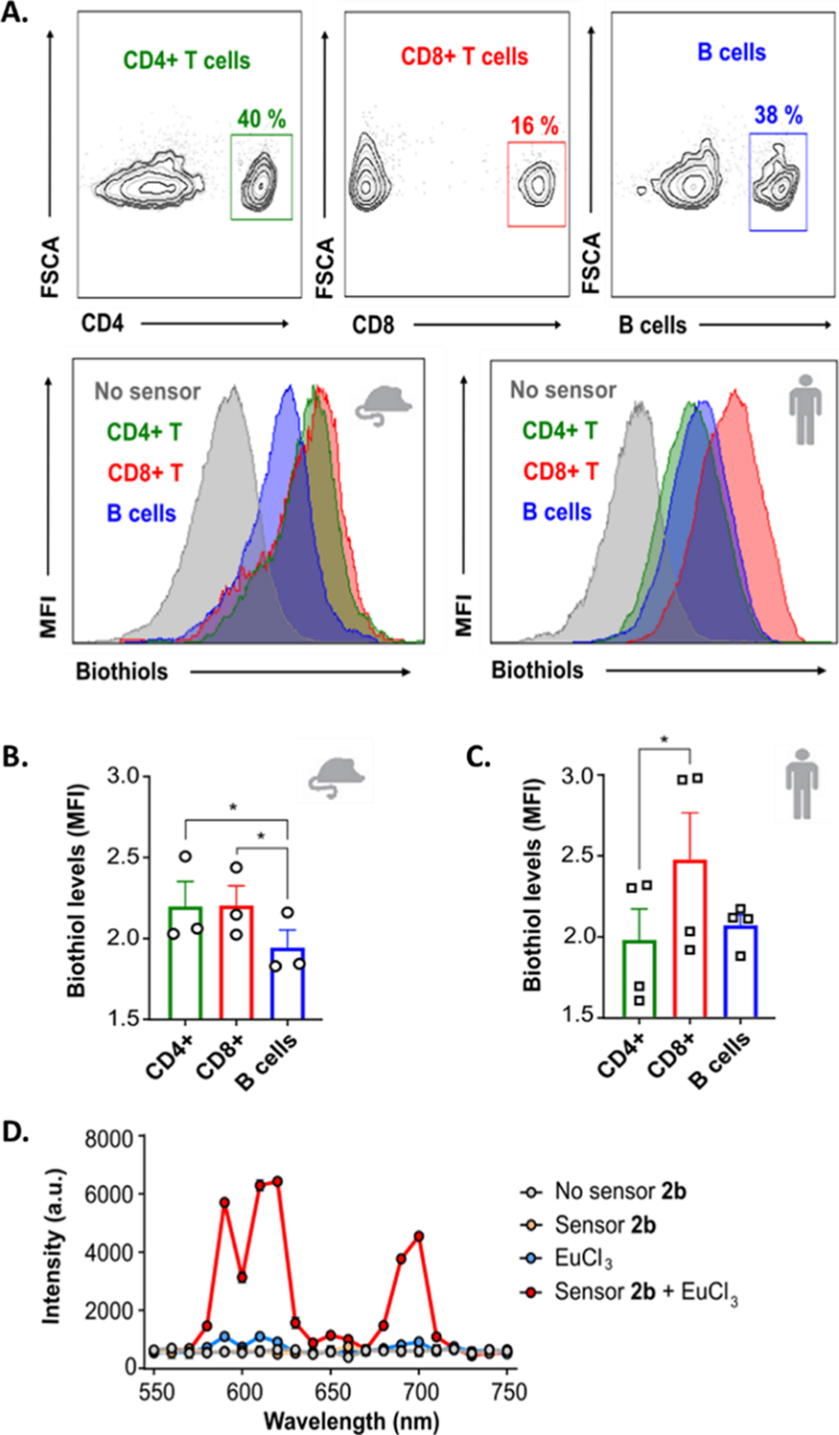
Study of the intracellular biothiol levels in murine and human immune cells with sensor **2b**. (A) Gating flow cytometry strategy to identify different cell sub-populations (CD4^+^ T cells in green, CD8^+^ T cells in red, and B cells in blue) and representative mean fluorescence intensities in the PacBlue channel either from murine or human cells (MFI, associated with intracellular biothiol levels); (B,C) flow cytometry quantification of the intracellular biothiol levels measured using the sensor at 25 μM concentration from different sub-populations of immune cells in mouse (MFI normalized to control without **2b**) (B) and human (C) (*n* = 3 animals/group or *n* = 4 human samples/group from three independent experiments, ANOVA with Tukey’s HSD *t*-test, *p < 0.05). (D) Time-resolved and time-gated luminescence spectra from splenocytes in the presence or absence of **2b**, EuCl_3_ (250 μM), or both (λ_ex_ = 320 nm).

To assess the applicability of **2b** to study intracellular GSH in primary cells, we focused on the immune system. We first utilized **2b** to evaluate the differences in biothiol levels at the baseline within different sub-populations (CD4^+^ T cells, CD8^+^ T cells, and B cells) of mouse splenocytes and human peripheral mononuclear cells (PBMCs) using flow cytometry. Of note, the sensor was not toxic and did not affect cell viability at a wide range of concentrations (0–50 μM) (Figure S10). To maximize the dynamic range of the measurements and capture differences within immune cell compartments, we used the sensor at 25 μM. Incubating the cells at this concentration, we were able to capture differences in the intracellular biothiol levels between CD4^+^ T cells, CD8^+^ T cells, and B cells, in both murine and human cells (Figure 6). Whereas we observed similar mouse intracellular biothiol levels in CD4^+^ and CD8^+^ T cells, B cells showed significantly lower levels (Figure 6A,B), suggesting that these cells might have lower baseline metabolic rates. Interestingly, we observed a different distribution in PBMCs, with baseline biothiol levels of CD4^+^ T cells and human B cells being similar, and higher levels in CD8^+^ T cells (Figure 6C). These results confirm that **2b** can be used in combination with flow cytometry to capture differences in intracellular biothiol levels within primary immune cell types.

Based on our previous outcomes, where **2b** also acted as a europium antenna after reacting with biothiols, we applied this alternate version of the sensor to study changes in immune cell intracellular biothiol levels, in this case using a time-resolved and time-gated intensity analysis adapted to detect the long luminescence lifetime of Eu^3+^. We cultured splenocytes from wild-type mice and studied the time-resolved and time-gated luminescence spectra between 550 and 750 nm after adding either the **2b** biosensor (25 μM), europium (EuCl_3_ at 250 μM), or both (Figure 6D). As expected, we only observed changes in the luminescence intensity when the **2b** sensor was added together with europium with the detected emission bands perfectly matching those of the Eu^3+^ emission. This result indicates that the sensor was able to intracellularly sensitize europium luminescence, which could only happen if the sensor and the cation Eu^3+^ successfully entered the cells and reacted with intracellular biothiols. Once the conditions for the time-resolved and time-gated analysis on splenocytes were optimized, we studied the sensitized emission of Eu^3+^ in splenocytes in response to biothiol levels for 14 h. Europium luminescence reached peak levels at the beginning of the experiment, slowly decreasing with time (within hours) (Figure S11), indicating that the Eu-based version of the sensor is an option for biological questions in which an increased signal-to-noise ratio (SNR) is required.

### **2b** Captures GSH Dynamic Changes in T_REG_

Regulatory T cells (T_REG_) are one of the main mediators of central and peripheral tolerance^68−70^ and thus play a key role in autoimmune diseases, organ transplant rejection, and also anti-tumor immune responses.^68−70^ GSH is vital for T-cell effector function and proliferation and for preserving T_REG_ function,^22^ making the levels of this intracellular species in T cells a particularly relevant signal to monitor.

**Figure 7:**
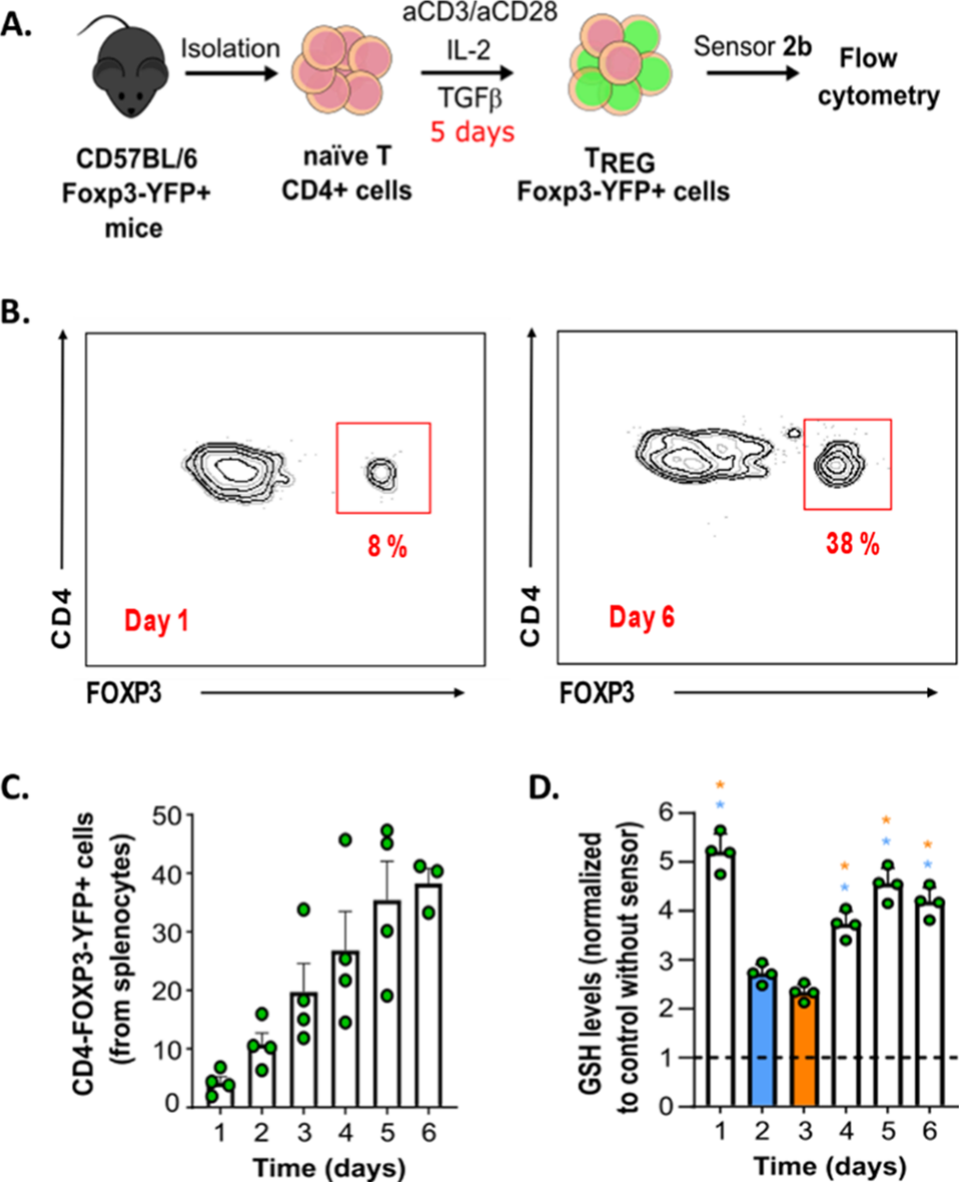
Study of biothiol metabolism in T_REG_ with sensor **2b**. (A) Schematic of the T_REG_ induction protocol used to monitor intracellular biothiol levels. (B) Representative scatter plots of flow cytometry analysis and quantification (C) of the number of CD4^+^ Foxp3-YFP^+^ cells in the culture at different days (1–6); and (D) flow cytometric quantification and statistical analysis of the intracellular biothiol levels in T_REG_ at different days (*n* = 4 animals/group from three independent experiments, ANOVA with Tukey’s HSD *t*-test, *p < 0.05, blue: comparison with *t* = 2 days, orange: comparison with *t* = 3 days).

We decided to test the ability of the **2b** sensor to measure GSH intracellular changes in T_REG_ induction cultures. To this aim, we isolated naïve splenic CD4^+^ T cells (defined as CD44^lo^ CD62L^hi^ purity > 95%) from C57BL/6-Foxp3-YFP mice and set up T_REG_ induction cultures by culturing them for 5 days with αCD3/αCD28 activating beads under T_REG_ polarizing conditions (IL-2 and TGFβ). In these mice, cells express a yellow fluorescent protein (YFP) fused to Foxp3, which can be detected as naïve T cells become T_REG_ (CD4^+^Foxp3^+^), allowing us to selectively study GSH levels in this sub-population. We monitored these levels in the culture daily by incubating with the sensor for 30 min and analyzing the cells by flow cytometry (Figure 7A). As it has been previously well established in these cultures, we observed an increase in the percentage of T_REG_ in the culture as a function of time which peaked at day 5 (Figure 7B).^71,72^ Interestingly, we observed a sharp increase in T_REG_ GSH levels at day 1, followed by a decrease at days 2 and 3 and then increasing again up to day 5 (Figure 7C). This result suggests that different metabolic processes act at different times in the process of becoming T_REG_.

## Conclusions

In conclusion, the results herein described show that the small non-fluorescent Michael acceptor **2b**, after its reaction with biothiols, becomes fluorescent and an efficient Eu^3+^ antenna, which self-assembles with the cation in water. This property makes **2b** a highly selective GSH biosensor, which can be monitored through either the increase of the fluorescence of the antenna or the luminescence of Eu^3+^, opening the possibility to multiplexing applications. We have demonstrated the potential of **2b** as a GSH biosensor to study murine and human cells of the immune system with flow cytometry (CD4^+^ T, CD8^+^ T, and B cells), and to monitor changes in their metabolism as naïve CD4^+^ T cells polarize to T_REG_. Together, these experiments constitute a proof-of-concept of the use of **2b** to monitor biothiols in immune cells, filling the gap for GSH-metabolic studies in flow cytometry to address biological questions and pave the way to its application to study clinical samples.

## Supporting Information Available

Detailed information on synthetic, NMR, fluorescence, and computational methods; time-resolved and flow cytometry methods;^1^H NMR and ^13^C NMR spectra of new compounds; tautomeric/rotameric structures; relative energies; calculated HOMA values; emission spectra; HPLC–MS analysis; fluorescence emission intensity; titration spectra; luminescence lifetimes; flow cytometric quantification; and time-resolved fluorescence kinetics (PDF)
