# Supplementary material for: Self-Assembled Lanthanide Antenna Glutathione Sensor for the Study of Immune Cells
Source: ACS Sens. 2022 Jan 15;7(1):322–30. doi: 10.1021/acssensors.1c02439 (PMC8805117; doi:10.1021/acssensors.1c02439)
Supplement: Supplementary file 1 — se1c02439_si_001.pdf [file se1c02439_si_001.pdf]

Supporting Information

Self-assembled lanthanide antenna glutathione sensor for the study of immune cells

Francisco Fueyo-González[a,c], Laura Espinar-Barranco[b], Rosario Herranz[a], Ibon Alkorta[a], Luis Crovetto[b], Miguel Fribourg[c], Jose Manuel Paredes[b], Angel Orte[b] and Juan A. González-Vera[a,b,]*

[a] *Instituto de Química Médica (CSIC). Juan de la Cierva 3, 28006 Madrid, Spain.*

[b] *Nanoscopy Laboratory. Departamento de Fisicoquímica, Unidad de Excelencia de Química Aplicada a Biomedicina y Medioambiente, Facultad de Farmacia, Universidad de Granada, Campus Cartuja, 18071, Granada, Spain. E-mail: gonzalezvera@ugr.es*

[c] *Department of Medicine, Translational Transplant Research Center, Immunology Institute, Icahn School of Medicine at Mount Sinai, New York, NY 10029 USA.*

<u>Table of Contents</u> **<u>PAGE</u>**

I. General methods S1
II. Experimental synthetic methods S2
III. Computational methods S3
IV. Spectroscopy Methods S7
V. Kinetic study S13
VI. Binding model and fitting parameters S14
VII. Cell Viability S15
VIII. Time-resolved luminescence assays S16
IX. Mice and Human subjects S16
X. Lymphocyte isolation from the spleen and PBMCs S17
XI. Flow cytometry, surface staining and biothiol levels S17
XII. *In vitro* T_{REG} induction S17
XIII. NMR spectra (^{1}H-NMR and ^{13}C-NMR) S18

I. General Methods

All reagents were of commercial quality. Solvents were dried and purified by standard methods. Analytical TLC was performed on aluminum sheets coated with a 0.2 mm layer of silica gel 60 F_{254}. Silica gel 60 (230-400 mesh) was used for flash chromatography. HPLC-MS was performed on a Sunfire C_{18} (4.6×50 mm, 3.5 µm) column at 30°C, with a flow rate of 1 mL/min and gradient of 0.1% of formic acid in CH_3CN (solvent A) in 0.1% of formic acid in H_2O (solvent B) was used as mobile phase. Electrospray in positive mode was used for ionization. NMR spectra were recorded using Varian Inova or Mercury 400, and Varian Unity 500 spectrometers. The NMR spectra assignments were based on COSY, HSQC, and HMBC spectra. High resolution mass spectra (HRMS) were recorded on an Agilent 6520 Q-TOF instrument with an ESI source. MW experiments were carried out in sealed vessels in a MW Emrys™ Synthesizer (Biotage AB), with transversal IR sensor for reaction temperature monitoring. UV-visible spectroscopy measurements were made at 25 Cº on a Lambda 35, Perkin Elmer, UV-vis spectrophotometer. Steady-state fluorescence emission spectra were performed at 25 ºC on a JASCO FP-8300 spectrofluorometer equipped with a 450 W xenon lamp for excitation. Lifetime experiments were acquired in a Varian Cary Eclipse Spectrofluorometer. Starna and Hellma quartz cuvettes of 1 cm path length and several volumes were employed.

II. Experimental Synthetic Methods

Synthesis of methyl 8-methoxy-2-oxo-1,2-dihydrocyclopenta[*de*]quinoline-3-carboxylate (2a). 2,3-Dichloro-5,6-dicyano-1,4-benzoquinone (DDQ, 900 mg, 3.95 mmol) was added to a solution of methyl 8-methoxy-2-oxo-1,2,4,5-tetrahydrocyclopenta[*de*]quinoline-3-carboxylate (**1a**)[1] (810 mg, 3,30 mmol) in toluene (25 mL), and the mixture was stirred and heated to reflux for 4 h. Then, the reaction mixture was evaporated to dryness and the residue was purified by flash chromatography, using 0-5% gradient of MeOH in CH_2Cl_2 as eluent to give the oxidized compound **2a** as a red solid (350 mg, 42%). M.p. 203 ºC. HPLC-MS (30-95% gradient of 0.1 % solution of A in B, 10 min) t_R = 2.32 min. ^{1}H-RMN ($CDCl_3$, 400 MHz) δ: 3.89 (s, 3H), 3.91 (s, 3H), 6.62 (d, 1H, J = 7.5 Hz), 6.82 (d, 1H, J = 5.3 Hz), 6.96 (d, 1H, J = 7.5 Hz), 7.05 (d, 1H, J = 5.3 Hz), 9.79 (s, 1H). ^{13}C-RMN ($CDCl_3$, 100 MHz) δ: 52.5, 56.4, 110.8, 118.3, 119.9, 120.1, 125.5, 1264, 131.5, 141.0, 147.7, 152.8, 161.7, 164.8. HRMS (ESI) *m/z*: Calcd. for $C_{14}H_{11}NO_4$ ($[M+H]^+$): 258.0761, Found: 258.0757.

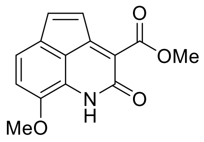

2a

[1] J. A. González-Vera, F. Fueyo-González, I. Alkorta, M. Peyressatre, M. C. Morris and R. Herranz, *Chem. Commun.*, 2016, **52**, 9652-9655.

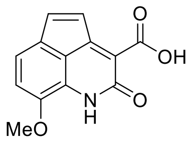

2b

Synthesis of 8-methoxy-2-oxo-1,2-dihydrocyclopenta[*de*]quinoline-3-carboxylic acid (2b). The methyl ester **2a** (200 mg, 0.77 mmol) was added to 2 N aqueous solution of NaOH (5 mL) and the mixture was heated to reflux for 30 min. Then, the solution was cooled and lyophilized, and the residue was purified by reverse flash chromatography using a KP-C18-HS cartridge and 0-10% gradient of CH_3CN in H_2O as eluent to give the free carboxylic acid **2b** as an orange-red solid (150 mg, 80 %). M.p. 274 ºC. HPLC-MS (5-95% gradient of 0.1 % solution of A in B, 10 min) t_R = 7.93 min.^{1}H-RMN (D_2O, 400 MHz) δ: 3.91 (s, 3H), 6.74 (d, 1H, *J* = 5.5 Hz), 6.77 (d, 1H, *J* = 7.5 Hz), 7.12 (2d, 2H, *J* = 5.5 Hz and *J* = 7.5 Hz).^{13}C-RMN (D_2O, 100 MHz) δ: 55.8, 109.6, 119.0, 119.1, 120.1, 124.4, 128.0, 130.8, 137.0, 144.2, 150.1, 166.3, 174.0. HRMS (ESI) *m/z*: Calcd. for $C_{13}H_9NO_4$ ($[M+H]^+$): 244.0604, Found: 244.0604.

III. Computational Methods

The geometry of the molecules in the singlet ground state (S_0) has been optimized with the B3LYP functional[2,3] and the 6-31+G(d,p) basis set.[4] Frequency calculations at the same computational level have been performed to confirm that the geometries obtained correspond to energetic minima. These geometries have been used as starting point for the optimization of the first excited single (S_1) and triplet (T_1) states. For the latter, TD-DFT[5] has been used. All the calculations have been performed with the Gaussian-16 program.

The aromaticity of the systems has been evaluated with the harmonic oscillator model of aromaticity (HOMA) parameter[6,7] as expressed in the following equation (S1).

$$HOMA = 1 - \frac{1}{n}\sum_1^n \propto_i (R_{opt} - R_i)^2 \qquad \text{(S1)}$$

The values used of R_{opt} and α were 1.388 and 257.7 for C-C bonds and 1.334 and 93.52 for C-N bonds, respectively.

---

[2] Becke, A. D. Density-functional thermochemistry. III. The role of exact exchange. *J. Chem. Phys.* **1993,** 98, 5648-52
[3] Lee, C.; Yang, W.; Parr, R. G. Development of the Colle-Salvetti correlation-energy formula into a functional of the electron density. *Phys. Rev. B* **1988,** 37, 785-789.
[4] Hariharan, P. C.; Pople, J. A. Influence of polarization functions on MO hydrogenation energies. *Theor. Chim. Acta* **1973,** 28, 213-22.
[5] Adamo, C.; Jacquemin, D. The calculations of excited-state properties with Time-Dependent Density Functional Theory. *Chem. Soc. Rev.* **2013,** 42, 845-856.
[6] Krygowski, T. M.; Cyranski, M. K. Structural Aspects of Aromaticity. *Chem. Rev.* **2001,** 101, 1385-1419.
[7] Krygowski, T. M. Crystallographic studies of inter- and intramolecular interactions reflected in aromatic character of π-electron systems. *J. Chem. Inf. Comput. Sci.* **1993,** 33, 70-8.

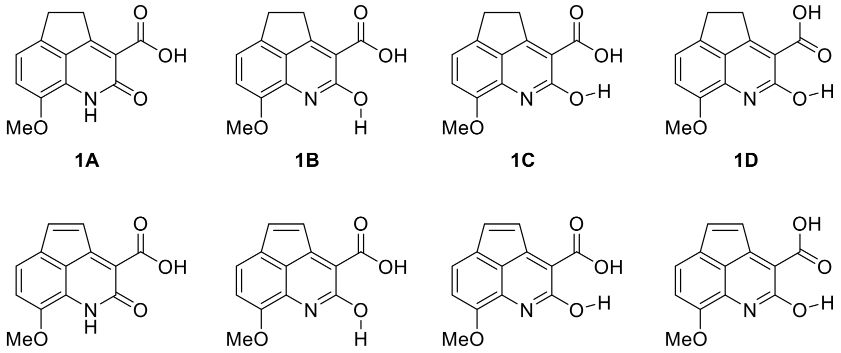

Figure S1. Tautomeric/rotameric structures considered in the calculations of the minimum energy structures of the S_0, S_1 and T_1 states for the free carboxylic acids **1b** and **2b**.

Table S1. Relative energies (E_{rel}, kJ mol^{-1}) of the **A-D** structures in the S_0, S_1 and T_1 states of **1b** and **2b**

Compd	Energy state	**A**	**B**	**C**	**D**
**1b**	S_0	0.0	29.3	30.0	16.2
	S_1	0.0	28.8	6.7	-20.6
	T_1	0.0	11.2	-0.9	-21.6
**2b**	S_0	0.0	26.0	26.0	9.6
	S_1	0.0	23.2	15.3	-1.6
	T_1	0.0	27.0	22.8	7.0

Table S2. Calculated HOMA values for periphery of the bicyclic quinolone ring of the **A-D** structures in the S_0, S_1 and T_1 energy states of **1b** and **2b**

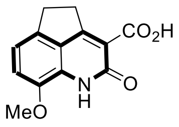

1b

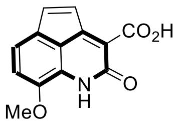

2b

Compd	Energy state	**A**	**B**	**C**	**D**
**1b**	S_0	0.58	0.79	0.74	0.73
	S_1	0.56	0.71	0.66	0.62
	T_1	0.62	0.64	0.64	0.66
**2b**	S_0	0.50	0.72	0.67	0.66
	S_1	0.43	0.62	0.57	0.56
	T_1	0.43	0.62	0.57	0.55

Table S3. Calculated HOMA values for periphery of the tricyclic ring of the 1,2-dihydrocyclopenta[*de*]quinoline system of **2b** in the **A-D** structures in the S_0, S_1 and T_1 energy states

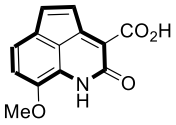

2b

Energy state	**A**	**B**	**C**	**D**
S_0	0.24	0.44	0.39	0.40
S_1	0.49	0.68	0.64	0.63
T_1	0.42	0.60	0.56	0.56

Table S4. ^{1}H NMR and ^{13}C NMR chemical shifts for **1b** and **2b**.

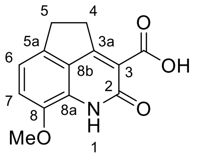

1b

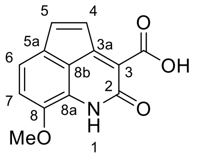

2b

Nucleus	**1b**[a] δ(pm)	**2b**[b] δ(pm)
4-H	3.51	6.74
5-H	3.15	7.12
6-H	6.93	7.12
7-H	6.98	6.77
2-C	169.4	174.0
C_2	167.6	166.3
C_3	116.4	119.0
C_{3a}	174.0	144.2
C_4	32.5	124.4
C_5	29.3	137.0
C_{5a}	137.8	130.8
C_6	114.7	120.1
C_7	112.0	109.6
C_8	149.6	150.1
C_{8a}	136.3	128.0
C_{8b}	130.9	119.1
OMe	55.5	55.8

[a]In DMSO-d_6. [b]In H_2O

IV. Spectroscopy Methods

a. Photophysical properties

Excitation and emission spectra of compounds were determined for 12 μM solutions in H_2O or CH_3CN. The spectra were recorded between 300 and 690 nm (0.5 nm increments and 0.1 s integration time) with excitation set at the appropriate excitation wavelength. Slit widths were set to 5 nm for excitation and to 5 nm for emission. All the spectra were corrected for background fluorescence by subtracting a blank scan of the solvent solution and spectrally corrected using certified fluorescence standards.

Fluorescence quantum yield determination. Fluorescence quantum yields (Φ_F) of quinolin-2(1*H*)-one derivatives **1b** and **2b** were determined in solvents of different polarity and calculated using quinine sulfate dihydrate (in 0.1M H_2SO_4) as reference.[8] Concentration of the sample and the reference was set to assure that the absorbance was less than 0.1 at identical excitation wavelengths. The following equation (S2) was used to calculate the quantum yield:

$$\Phi = \frac{I_x A_r n^2{}_x \Phi_r}{A_x I_r n^2{}_r} \qquad \text{(S2)}$$

where *x* and *r* denote the sample and standard, respectively, *A* is the absorption at the excitation wavelength, *I* is the integrated fluorescence intensity, and *n* is the refractive index of the solvent.

Fluorescence lifetimes determination. Lifetime experiments of lanthanide complexes of the addition products of GSH, Hcy and Cys to **2b** (**2b**-GSH, **2b**-Hcy and **2b**-Cys) were acquired in a Varian Cary Eclipse Spectrofluorometer at room temperature using the following conditions: excitation wavelength 320 nm; emission wavelength 545 nm for terbium or 615 nm for europium; excitation slit width 5.0 nm, emission slit width 5.0 nm; total decay time 15.0 ms; delay time 0.1 ms; gate time 0.2 ms; number of cycles 20; PMT detector voltage 600 V.

---

[8] (a) Wuerth, C.; Grabolle, M.; Pauli, J.; Spieles, M.; Resch-Genger, U. *Nat. Protoc.*, **2013**, *8*, 1535-1550 (b) Brouwer, A. M. *Pure Appl. Chem.*, **2011**, *83*, 2213-2228; (c) Rurack, K.; Spieles. M. *Anal. Chem.*, **2011**, *83*, 1232-1242.

b. Lanthanide luminescence sensitization and 2b reactivity studies

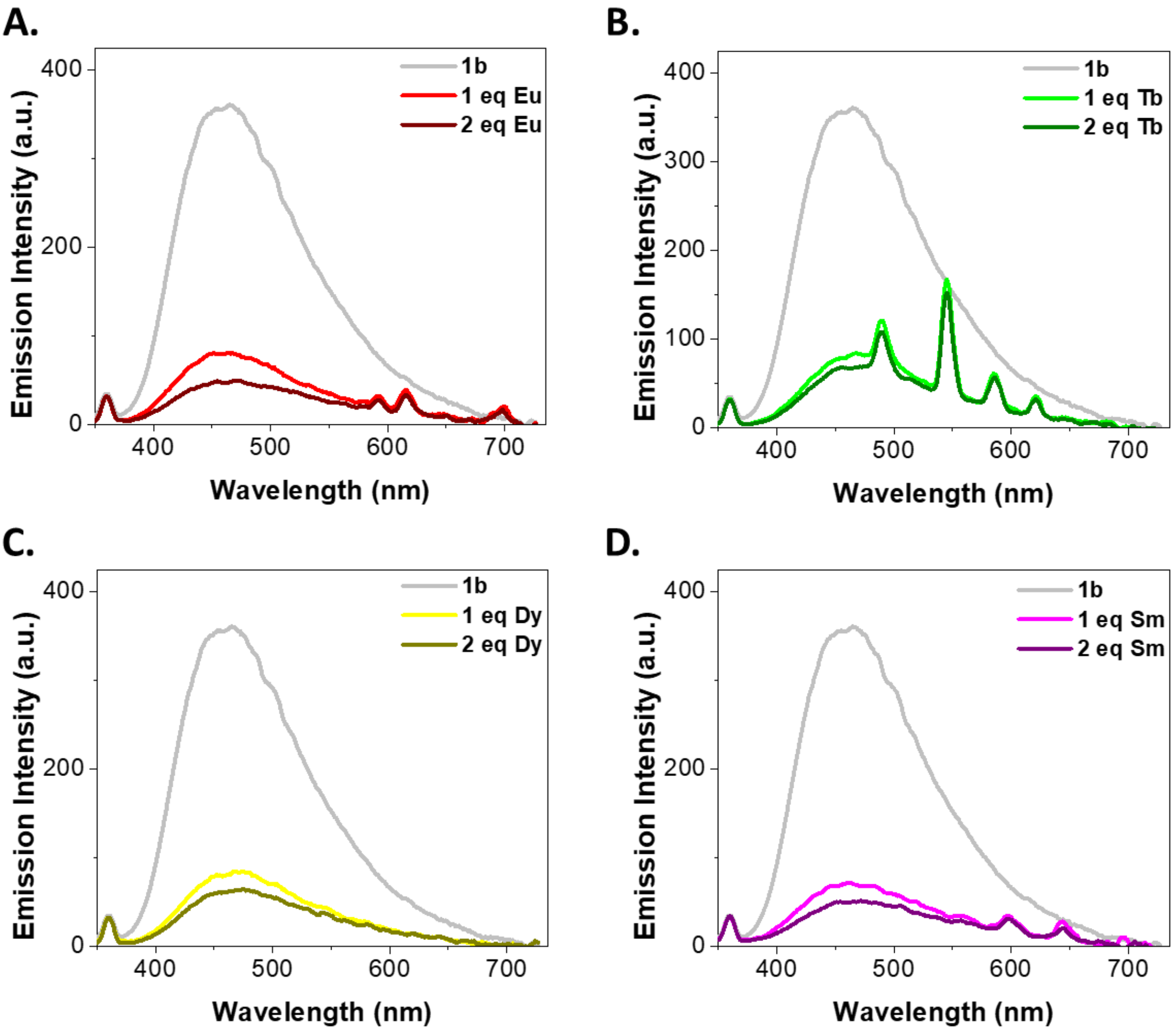

Figure S2. Emission spectra of **1b** (5 µM in Hepes buffer 50 mM, pH 7.4) and after addition of 1 and 2 equivalents of (A) $EuCl_3$, (B) $TbCl_3$, (C) $DyCl_3$ and (D) $SmCl_3$.

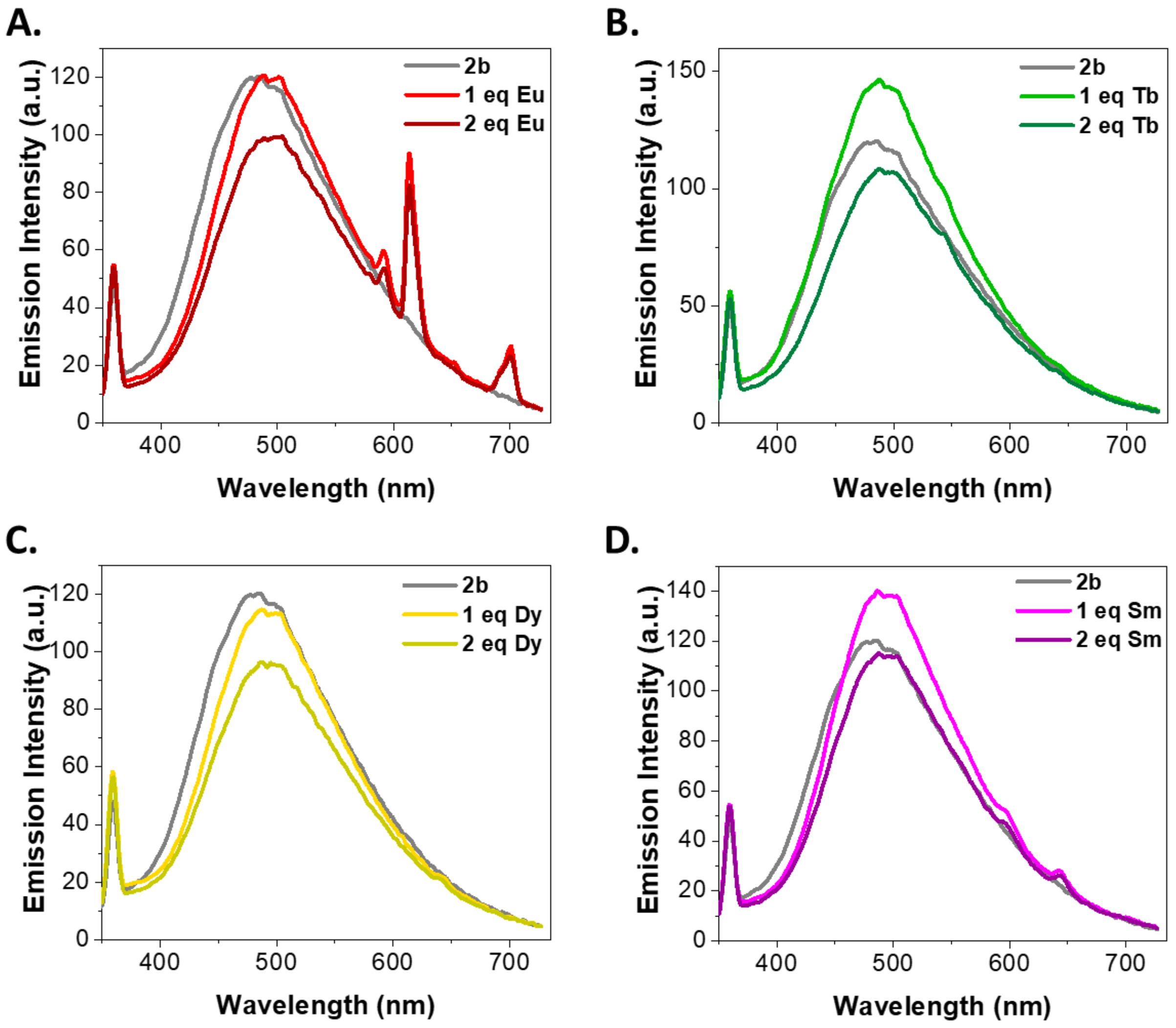

Figure S3. Emission spectra of **2b** (5 µM in Hepes buffer 50 mM, pH 7.4) and after addition of 1 and 2 equivalents of (A) $EuCl_3$, (B) $TbCl_3$, (C) $DyCl_3$ and (D) $SmCl_3$.

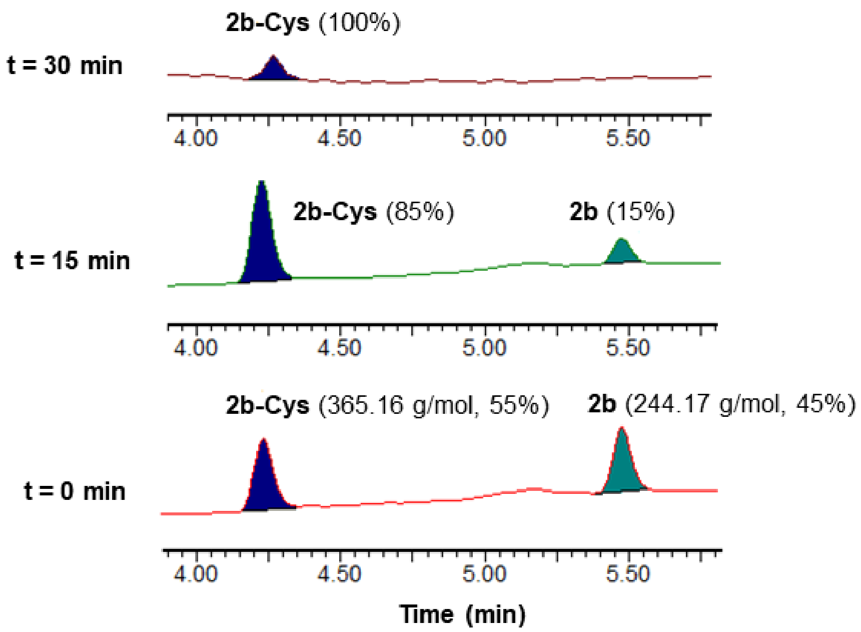

Figure S4. HPLC-MS analysis of **2b** (5 µM) after the addition of Cys (500 µM) in Hepes buffer pH 7.4.

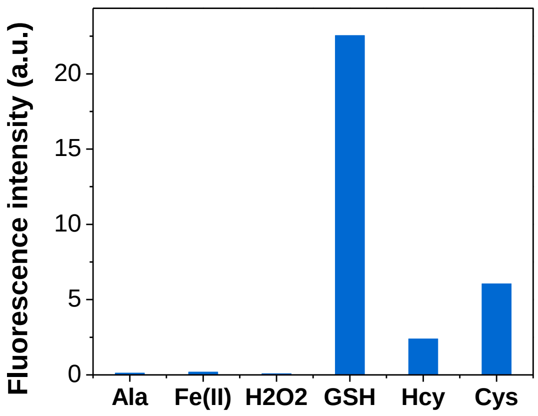

Figure S5. Fluorescence emission intensity of **2b** at 450 nm (5 µM in Hepes buffer 50 mM, pH 7.4) after addition of 100 equivalents of GSH and the possible interferents Ala, Fe^{2+}, H_2O_2.

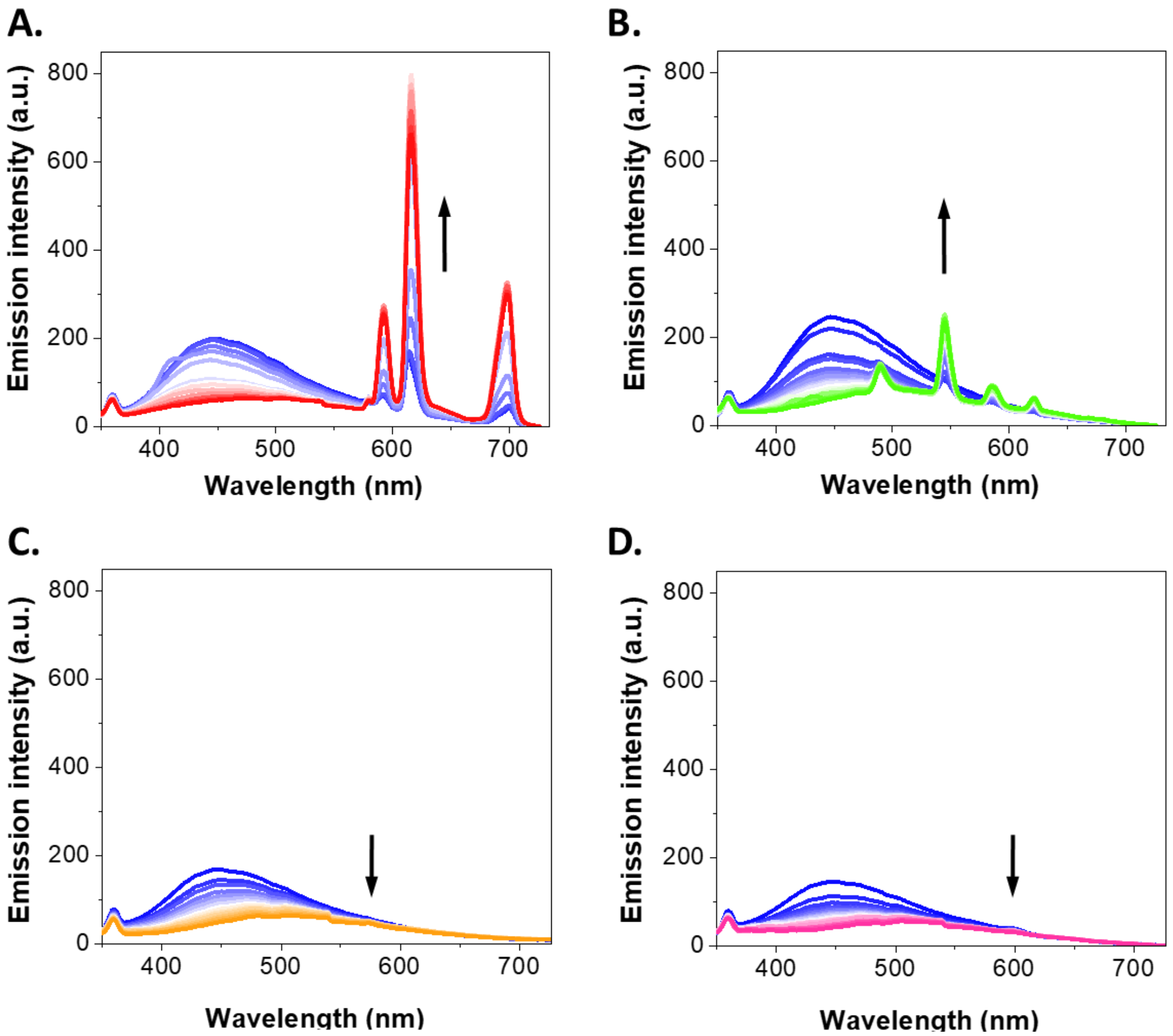

Figure S6. Titration spectra of **2b**-GSH (5 µM in Hepes buffer 50 mM, pH 7.4) with increasing equivalents (2–1000), indicated by arrows, of (A) $EuCl_3$, (B) $TbCl_3$, (C) $DyCl_3$ and (D) $SmCl_3$. Only with Eu^{3+} and Tb^{3+} the energy transfer was sufficiently effective to detect the lanthanide emission.

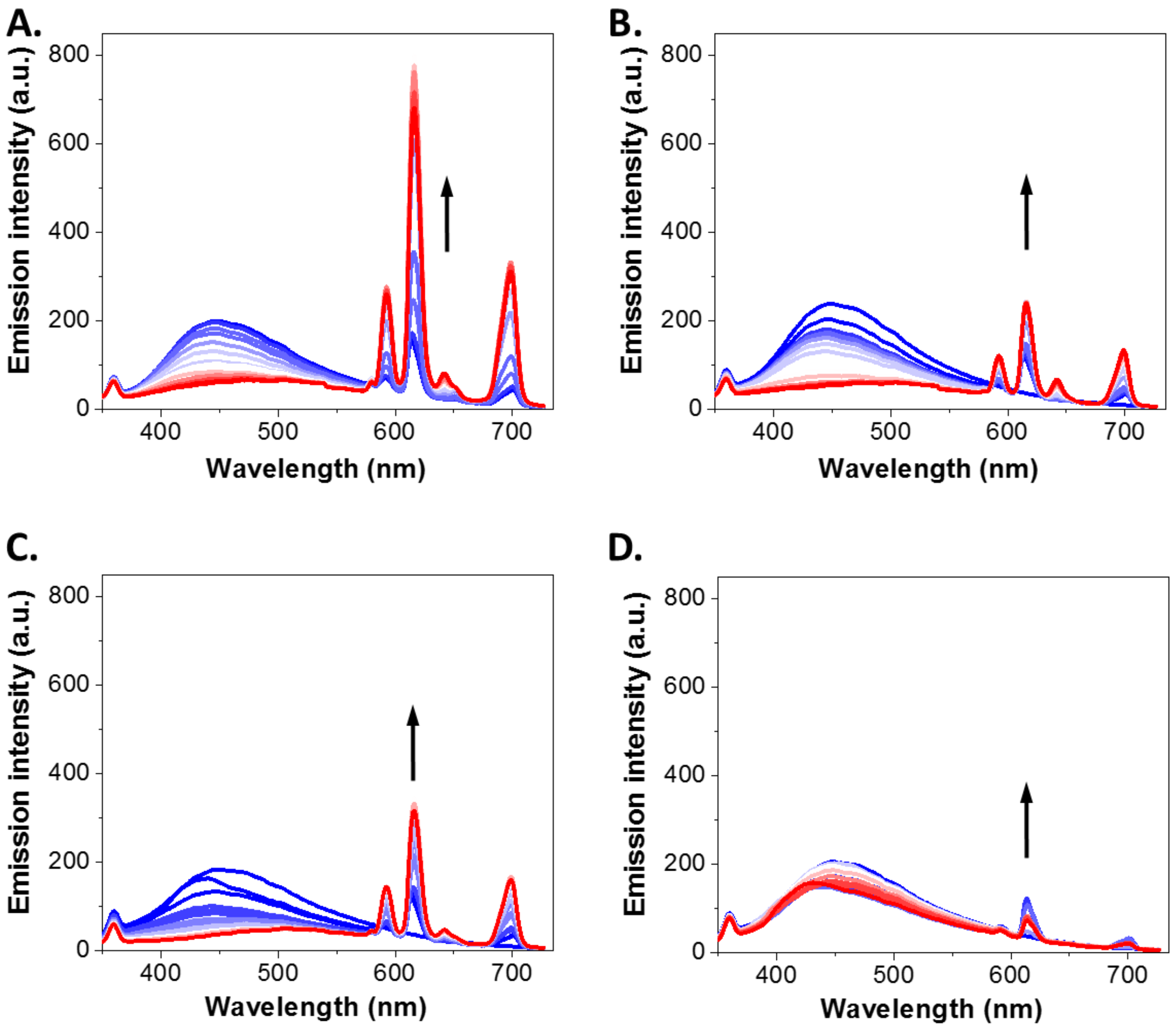

Figure S7. Titration spectra of (A) **2b**-GSH, (B) **2b**-Hcy, (C) **2b**-Cys and (D) **2b**-H_2S (5 μM, λ_{ex} = 320 nm) with increasing molar concentration of $EuCl_3$ (0.025-6.0 mM, increase indicated by arrows).

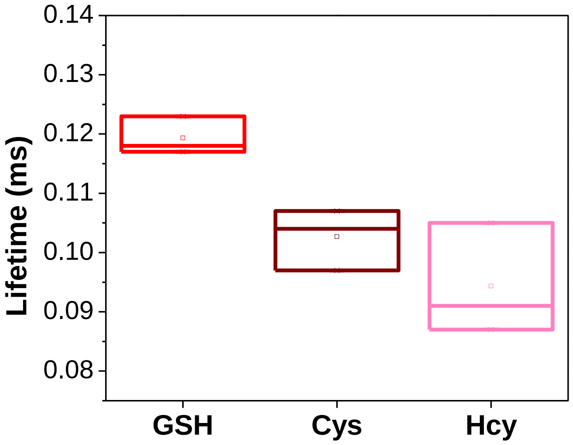

Figure S8. Luminescence lifetimes of lanthanide complexes **2b**-GSH, **2b**-Hcy and **2b**-Cys (5 µM in Hepes buffer 50 mM, pH 7.4) after addition of 100 equivalents of $EuCl_3$. Boxes correspond to the 25 and 75%, with a horizontal bar indicating the average value.

V. Kinetic study

In the kinetic study, determined the rate law from the experimental was determined data using the differential method.

Since we worked with constant concentrations of the sensor, we can focus on the reaction order with respect to GSH, using the following logarithmic equation (S3):

$$\log v_0 = \log k_{obs} + n \log[GSH]_0 \qquad \text{(S3)}$$

One way to obtain the data is to plot the initial rate of the reaction (v_0) with different initial $[GSH]_0$. This technique is known as the initial-rate method. A plot of log v_0 against log $[GSH]_0$ would be a line with a slope equal to the reaction order with respect to GSH (Figure S9). The observed rate constant, which would include components depending on the concentration of the sensor, can be determined by the intercept. To obtain the data, we used different concentrations of GSH (5, 25, 50 and 500 µM) in the presence of $EuCl_3$ (1.5 mM), in Hepes buffer (50 mM, pH 7.4), keeping the **2b** concentration constant (5 µM). The analysis of the kinetics has been carried out using Origin 8.5, (OriginLab Corp., MA, USA)

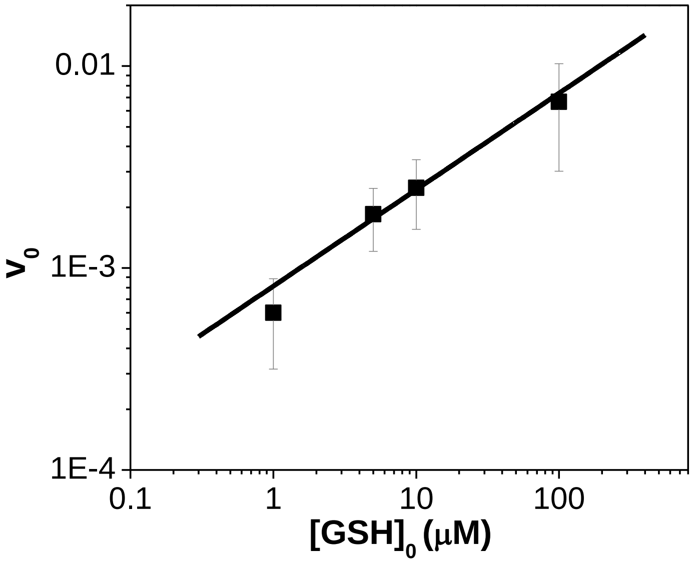

Figure S9. Representation of the logarithm of the initial slope of the kinetics versus the logarithm of the GSH concentration. The data show the average of three different kinetic curves. The error bars represent the standard deviation. The line represents the linear fitting to the following equation: $\log v_0 = -3.50 + 0.51 \log[GSH]_0$.

VI. Binding model and fitting parameters

The titration curves of lanthanide complexes **2b**-GSH, **2b**-Hcy and **2b**-Cys with europium (Figure 3D) were satisfactorily fitted to a dose-response binding isotherm, with adjustable Hill slope (*n*) (equation S4):

$$F = F_{min} + (F_{max} - F_{min}) \frac{[Ln]^n}{K^n + [Ln]^n} \qquad (S4)$$

where *F*, F_{min} and F_{max} accounts, respectively, for current, minimum and maximum luminescence intensity at lanthanide emission wavelength; *K*, the apparent binding constant of the antenna-lanthanide complex; [Ln], the concentration of lanthanide; *n*, the Hill slope.

VII. Cell Viability

We incubated mouse splenocytes (Figures S10A and S10B) or human PBMCs (Figure S10C) with either compound **2b** (Figures S1A and S1C) or $EuCl_3$ (Figure S10B) for 1 hour in PBS at 37 °C. We then washed the cells and stained them with fluorescent Fixable viability dye eFluor 780 (eBioscience, USA) as detailed in the manufacturer's protocol, and monitoring on a 3-laser Canto flow cytometer (BD bioscience) and analyzed the cells using FCS express software.

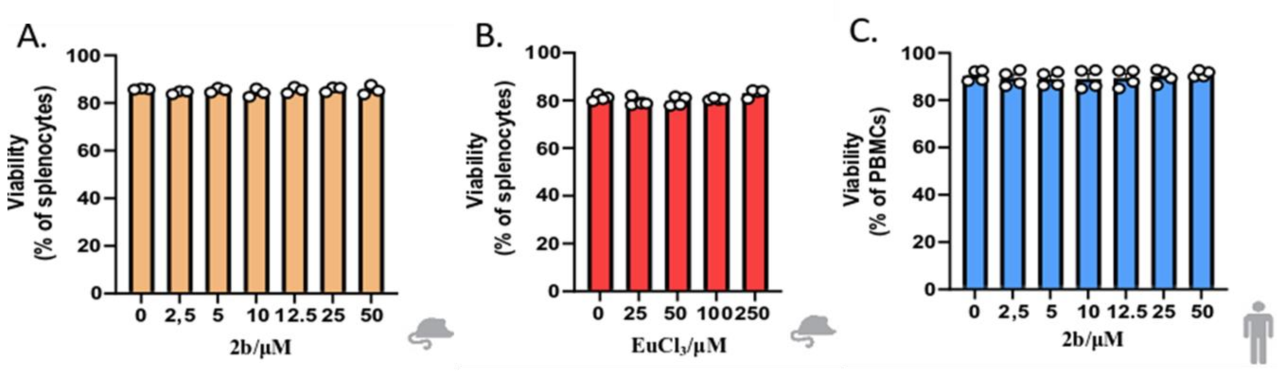

Figure S10. Flow cytometric quantification of the cell viability in mouse splenocytes (A, B) and human PBMCs (C). A. Mouse splenocytes incubated with **2b** at concentrations ranging from 0-50 μM; B. Mouse splenocytes incubated with $EuCl_3$ at concentrations ranging from 0-250 μM; C. Human PBMCs incubated with **2b** at concentrations ranging from 0-50 μM; (n=3 (A) or 4 (B) animals/group or n=4 (C) human samples/group from three independent experiments, ANOVA with Tukey HSD t-test, * $p<0.05$).

VIII. Time-resolved luminescence assays

Luminescent assays were performed in 96 well plates in 200 µL 50 mM potassium phosphate (50 mM KH_2PO_4/K_2PO_4, pH 7.4) 150 mM NaCl, using a SpectraMax M2 spectrofluorimeter. Splenocytes were cultured in PBS with the sensor **2b** (25 µM) and $EuCl_3$ (250 µM) added at the same time, and then excited at 320 nm. The emission signal was acquired at 620 nm unless stated otherwise (measurement start 50 µs; measurement end 850 µs; 20 flashes per read). Data analysis was performed using Prism software. Experiments were performed in triplicate, and error bars indicate standard deviation from average.

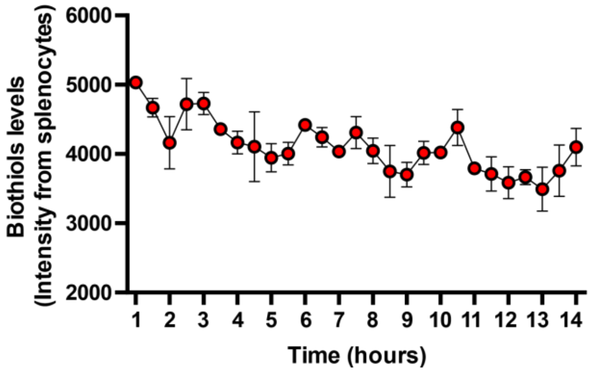

Figure S11. Time resolved-fluorescence kinetics of europium luminescence overtime (λ_{ex}= 320 nm, λ_{em}=620 nm, 14 h) in mouse splenocytes.

IX. Mice and human samples

Male and female C57BL/6-*Foxp3*-YFP and Wild type B6 mice were purchased from The Jackson Laboratory (stocks #016959 and #000651, respectively) and bred at mouse facilities of the Icahn School of Medicine at Mount Sinai. Mice were aged 8 weeks at the start of the experiment. Data depicted in the figures include male and female mice as we used both male and female mice for T_{REG} induction experiments. This study was carried out in strict accordance with the recommendations in the Guide for the Care and Use of Laboratory Animals and under the protocol (IACUC-2018-2) approved by the Institutional Animal Care and Use Committee of the Icahn School of Medicine at Mount Sinai of the USA. All animals were housed in the animal facilities at the Icahn School of Medicine at Mount Sinai.

For experiments in human cells we used peripheral blood mononuclear cells (PBMCs) from buffy coats obtained from Fully anonymous deidentified donors to the New York Blood Bank of the USA, and thus were determined by the institution not to constitute human subjects research.

X. Lymphocyte isolation from the spleen and PBMCs

Spleens were harvested in PBS and mechanically disaggregated through a mesh strainer (70 μm) with the aid of the back of a syringe plunger. PBS was decanted after centrifugation and red blood cells lysed (ACK lysis buffer, 4 min at room temperature, Roche). Cells were then resuspended in PBS and filtered again through a 70 μm nylon mesh. The isolated single-cell suspension of enriched either spleen or PBMCs leukocytes was processed for different assays or used to isolate naïve $CD4^+$ T cells naïve T cells using magnetic separation (EasySep™ Mouse or Human Naïve $CD4^+$ T Cell Isolation Kit, STEMCELL Kit) for T_{REG} induction cultures.

XI. Flow cytometry, surface staining and biothiol levels

Cells were evaluated for surface antigen expression (CD4, PE-cyanine7 Anti-mouse CD4 (GK 1.5), TONBO biosciences, USA) and intracellular biothiol levels culturing the cells either with the sensor at 25 μM or the sensor with $EuCl_3$ (250 μM) at the same time in the incubator for 30 minutes, following washes in PBS or a buffer consisting of 2% rat serum 2 mM EDTA. Data were acquired on a 3-laser Canto flow cytometer (BD bioscience) and analyzed using FCS express software.

XII. *In vitro* T_{REG} induction

In vitro T_{REG} induction either mouse naïve $CD4^+$ T cells or Human peripheral blood mononuclear cells (PBMCs) were isolated from spleens or buffy coats by Ficoll density gradient centrifugation (Histopaque, SigmaAldrich) at 490g. Naïve $CD4^+$ T cells were enriched from murine splenocytes (CD44loCD62hi) or human peripheral blood mononuclear cells (CD45RA+CD45RO-) (EasySep™ Mouse Naïve $CD4^+$ T Cell Isolation Kit, EasySep™ Human Naïve $CD4^+$ T Cell Isolation Kit, respectively, STEMCELL Technologies) and their purity checked by flow cytometry. Mouse cultures: 200,000 naïve $CD4^+$ T cells were incubated with IL-2 (2.75 ng/ml, Peprotech), TGFβ (0.7 ng/ml, Peprotech) and stimulated with either αCD3/αCD28 (15 μl/million cells, Gibco) (polyclonal). Human cultures: 200,000 naïve $CD4^+$ T cells were cultured with IL-2 (100 U/ml, BD Pharmingen), TGFβ (3 ng/ml, Peprotech) and stimulated with αCD3/αCD28 (15 μl/million cells, Gibco).

XIII. NMR SPECTRA (^{1}H-NMR AND ^{13}C-NMR) OF 2a AND 2b

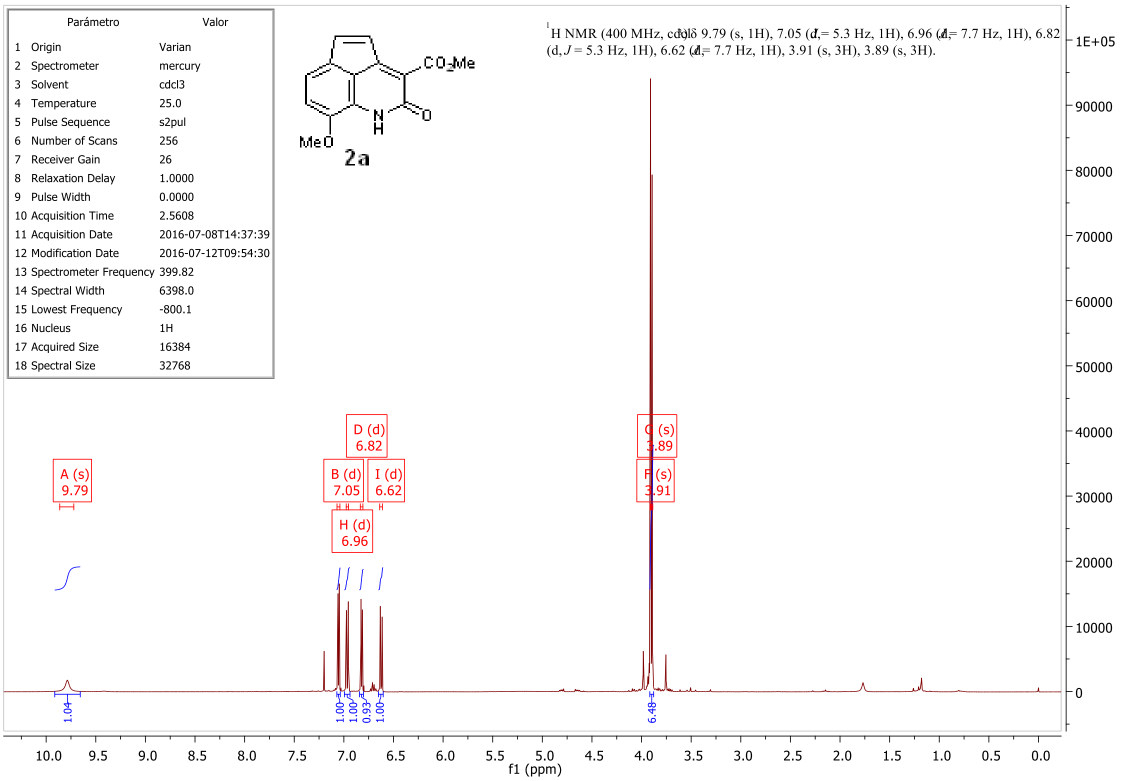
Parámetro Valor
1 Origin Varian
2 Spectrometer mercury
3 Solvent cdcl3
4 Temperature 25.0
5 Pulse Sequence s2pul
6 Number of Scans 256
7 Receiver Gain 26
8 Relaxation Delay 1.0000
9 Pulse Width 0.0000
10 Acquisition Time 2.5608
11 Acquisition Date 2016-07-08T14:37:39
12 Modification Date 2016-07-12T09:54:30
13 Spectrometer Frequency 399.82
14 Spectral Width 6398.0
15 Lowest Frequency -800.1
16 Nucleus 1H
17 Acquired Size 16384
18 Spectral Size 32768
CO2Me
N
H
O
MeO
2a
1H NMR (400 MHz, cdcl3) δ 9.79 (s, 1H), 7.05 (d, J = 5.3 Hz, 1H), 6.96 (d, J = 7.7 Hz, 1H), 6.82 (d, J = 5.3 Hz, 1H), 6.62 (d, J = 7.7 Hz, 1H), 3.91 (s, 3H), 3.89 (s, 3H).
A (s) 9.79
B (d) 7.05
D (d) 6.82
H (d) 6.96
I (d) 6.62
C (s) 3.89
F (s) 3.91
1.04
1.00
1.00
0.93
1.00
6.48
f1 (ppm)

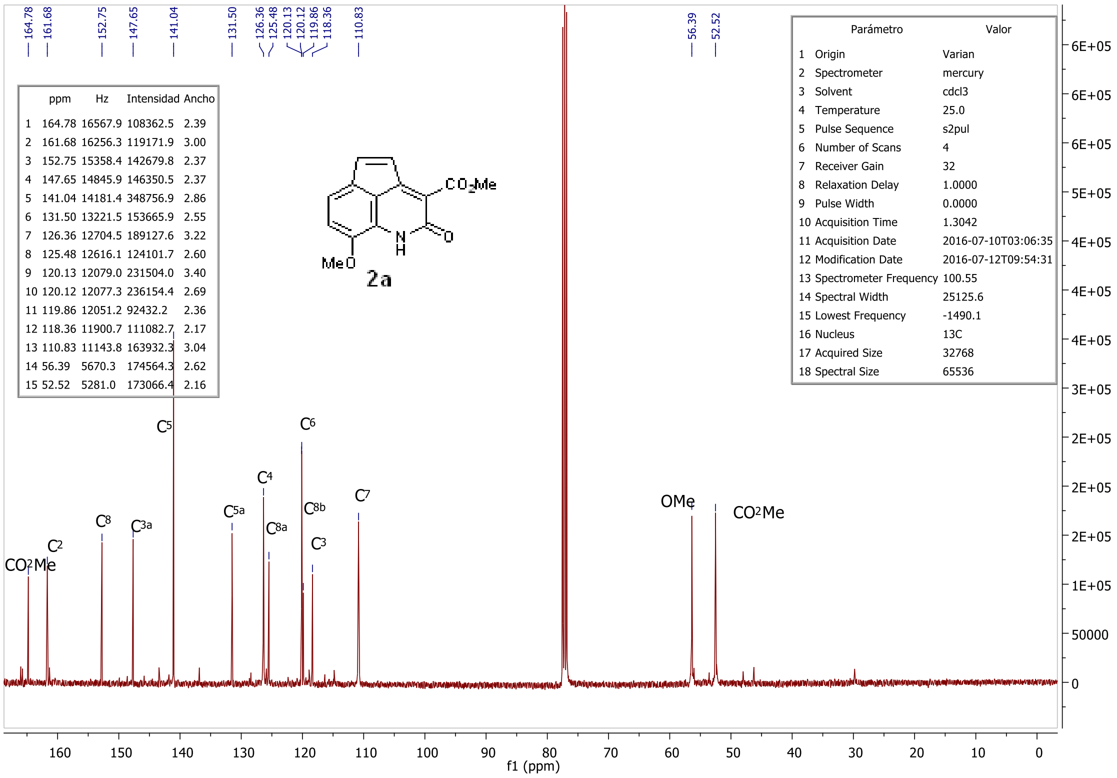
164.78
161.68
152.75
147.65
141.04
131.50
126.36
125.48
120.13
120.12
119.86
118.36
110.83
56.39
52.52
ppm Hz Intensidad Ancho
1 164.78 16567.9 108362.5 2.39
2 161.68 16256.3 119171.9 3.00
3 152.75 15358.4 142679.8 2.37
4 147.65 14845.9 146350.5 2.37
5 141.04 14181.4 348756.9 2.86
6 131.50 13221.5 153665.9 2.55
7 126.36 12704.5 189127.6 3.22
8 125.48 12616.1 124101.7 2.60
9 120.13 12079.0 231504.0 3.40
10 120.12 12077.3 236154.4 2.69
11 119.86 12051.2 92432.2 2.36
12 118.36 11900.7 111082.7 2.17
13 110.83 11143.8 163932.3 3.04
14 56.39 5670.3 174564.3 2.62
15 52.52 5281.0 173066.4 2.16
CO2Me
N
H
O
MeO
2a
Parámetro Valor
1 Origin Varian
2 Spectrometer mercury
3 Solvent cdcl3
4 Temperature 25.0
5 Pulse Sequence s2pul
6 Number of Scans 4
7 Receiver Gain 32
8 Relaxation Delay 1.0000
9 Pulse Width 0.0000
10 Acquisition Time 1.3042
11 Acquisition Date 2016-07-10T03:06:35
12 Modification Date 2016-07-12T09:54:31
13 Spectrometer Frequency 100.55
14 Spectral Width 25125.6
15 Lowest Frequency -1490.1
16 Nucleus 13C
17 Acquired Size 32768
18 Spectral Size 65536
C5
C6
C4
C5a
C8b
C7
C8
C3a
C8a
C3
C2
CO2Me
OMe
CO2Me
f1 (ppm)

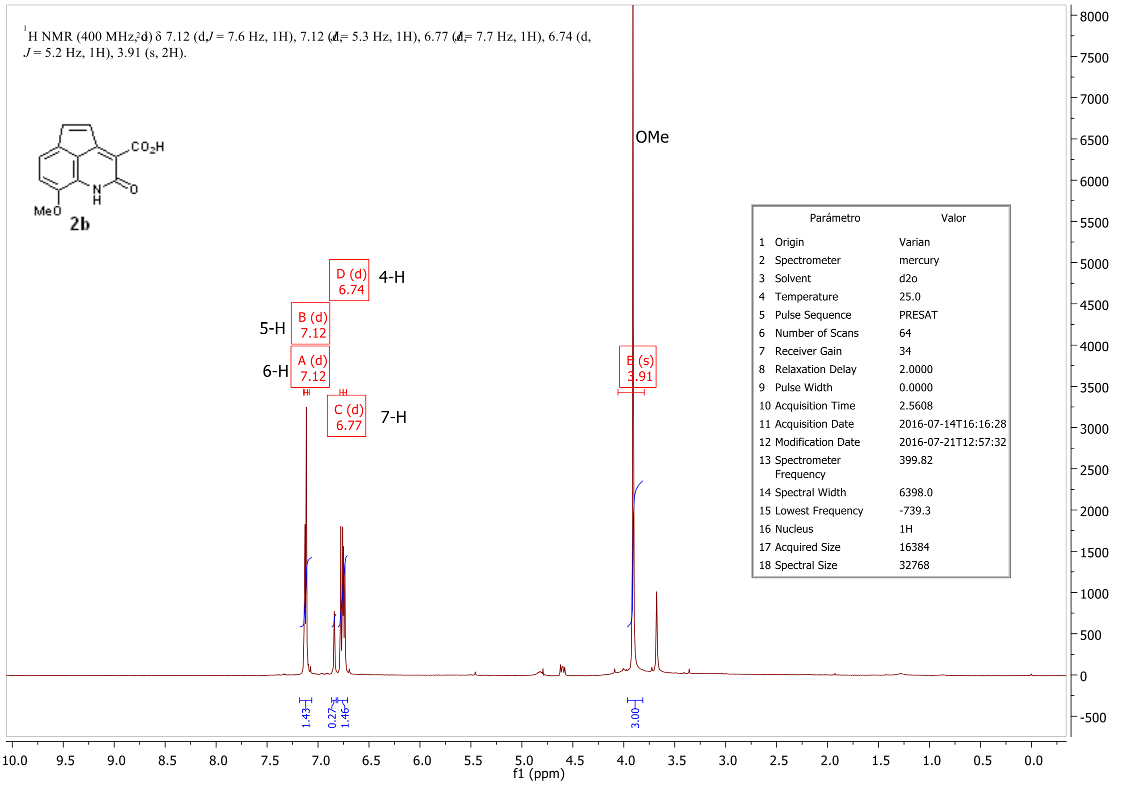
1H NMR (400 MHz, d2o) δ 7.12 (d, J = 7.6 Hz, 1H), 7.12 (d, J = 5.3 Hz, 1H), 6.77 (d, J = 7.7 Hz, 1H), 6.74 (d, J = 5.2 Hz, 1H), 3.91 (s, 2H).
CO2H
MeO
N
H
O
2b
OMe
D (d) 6.74
4-H
B (d) 7.12
5-H
A (d) 7.12
6-H
C (d) 6.77
7-H
E (s) 3.91
Parámetro Valor
1 Origin Varian
2 Spectrometer mercury
3 Solvent d2o
4 Temperature 25.0
5 Pulse Sequence PRESAT
6 Number of Scans 64
7 Receiver Gain 34
8 Relaxation Delay 2.0000
9 Pulse Width 0.0000
10 Acquisition Time 2.5608
11 Acquisition Date 2016-07-14T16:16:28
12 Modification Date 2016-07-21T12:57:32
13 Spectrometer Frequency 399.82
14 Spectral Width 6398.0
15 Lowest Frequency -739.3
16 Nucleus 1H
17 Acquired Size 16384
18 Spectral Size 32768
1.43
0.27
1.46
3.00
f1 (ppm)

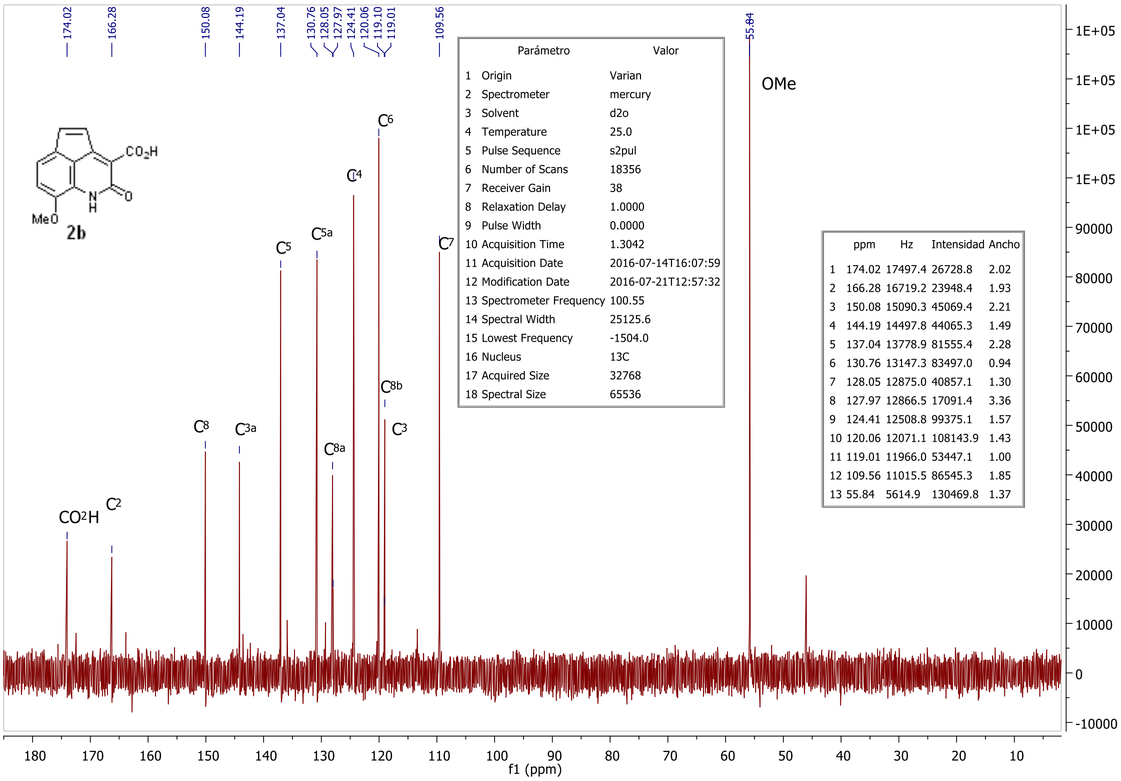
174.02
166.28
150.08
144.19
137.04
130.76
128.05
127.97
124.41
120.06
119.10
119.01
109.56
55.84
CO2H
MeO
N
H
O
2b
OMe
C6
C4
C5
C5a
C7
C8b
C3
C8
C3a
C8a
CO2H
C2
Parámetro Valor
1 Origin Varian
2 Spectrometer mercury
3 Solvent d2o
4 Temperature 25.0
5 Pulse Sequence s2pul
6 Number of Scans 18356
7 Receiver Gain 38
8 Relaxation Delay 1.0000
9 Pulse Width 0.0000
10 Acquisition Time 1.3042
11 Acquisition Date 2016-07-14T16:07:59
12 Modification Date 2016-07-21T12:57:32
13 Spectrometer Frequency 100.55
14 Spectral Width 25125.6
15 Lowest Frequency -1504.0
16 Nucleus 13C
17 Acquired Size 32768
18 Spectral Size 65536
ppm Hz Intensidad Ancho
1 174.02 17497.4 26728.8 2.02
2 166.28 16719.2 23948.4 1.93
3 150.08 15090.3 45069.4 2.21
4 144.19 14497.8 44065.3 1.49
5 137.04 13778.9 81555.4 2.28
6 130.76 13147.3 83497.0 0.94
7 128.05 12875.0 40857.1 1.30
8 127.97 12866.5 17091.4 3.36
9 124.41 12508.8 99375.1 1.57
10 120.06 12071.1 108143.9 1.43
11 119.01 11966.0 53447.1 1.00
12 109.56 11015.5 86545.3 1.85
13 55.84 5614.9 130469.8 1.37
f1 (ppm)
